# Phosphorylation of MdWRKY70L by MdMPK6/02G mediates reactive oxygen accumulation to regulate apple fruit senescence

**DOI:** 10.1111/pbi.70067

**Published:** 2025-03-24

**Authors:** Hui Wang, Yuchen Feng, Shuhui Zhang, Lulong Sun, Peng Yan, Yifeng Feng, Zhengyang Zhao

**Affiliations:** ^1^ State Key Laboratory of Crop Stress Biology for Arid Areas, College of Horticulture Northwest A&F University Yangling China

**Keywords:** apple, fruit senescence, *MdWRKY70L*, phosphorylated, ROS accumulation

## Abstract

Apple (*Malus domestica* Borkh.) is a globally significant crop and a vital dietary component worldwide. During ripening, apples exhibit a longitudinal gradient, ripening first at the stalk cavity and extending towards the calyx concave. When the fruit is harvested at the right time or later, the stalk cavity of many varieties often shows over‐ripening, that is, premature senescence such as peel browning, which diminishes fruit quality. This study examines the natural senescence process in 6‐year‐old ‘Ruixue’ apples by screening transcriptome data to uncover senescence‐related genes and validate their molecular functions. Our analysis of antioxidant capacity and reactive oxygen species (ROS) in different peel regions revealed that malondialdehyde (MDA), hydrogen peroxide (H_2_O_2_), and superoxide anion ($O_{2}^{-\cdot }$) levels increased with senescence, where ROS‐scavenging enzyme activity was notably reduced, especially in the stalk cavity (compared with the fruits in the stalk cavity at 120 days, the activities of SOD, POD, and CAT in stalk cavity of fruits at 205 days were significantly decreased in 65.4%, 82.7%, and 91.1%, respectively). Transcriptome clustering and enrichment analyses across developmental stages revealed *MdWRKY70L*, *MdSAG101*, and *MdZAT12* as key regulators of peel senescence. MdWRKY70L could interact with *MdSAG101*/*MdZAT12* both *in vivo* and *in vitro*, thereby mediating ROS accumulation in the peel and accelerating the fruit senescence process. Further *in vitro* and *in vivo* studies demonstrated that MdWRKY70L is phosphorylated at Ser199 by MdMPK6/02G, enhancing MdWRKY70L protein stability and promoting peel senescence. These findings offer insights for developing strategies to delay fruit senescence and improve postharvest quality control.

## Introduction

Apple (*Malus domestica* Borkh.) ranks among the top economically valuable crops globally. Its fruits are rich in essential vitamins, antioxidants, and cellulose, which are crucial components of the human diet and key to the global fruit trade (Wang *et al*., 2023; Zhao *et al*., 2020). However, large‐scale production and concentrated harvest times often result in delayed harvesting, pushing apples rapidly into natural senescence and triggering programmed cell death (Tian *et al*., 2013; Wang *et al*., 2023). Research on tomatoes has unveiled that fruit maturation follows a longitudinal gradient, beginning at the peduncle and advancing towards the stalk cavity, with coordinated genetic, hormonal, and metabolic changes along this axis (Huang *et al*., 2022; Shinozaki *et al*., 2018). Our previous investigation observed similar patterns in apples, where ripening initiates in the stalk cavity. By full maturity, the stalk cavity begins senescing, showing browning spots at the stalk cavity during pre‐harvest and expanding to the whole surface of the fruits. Meanwhile, the stalk cavity and other expanded parts change from pale brown to brown and dark brown, which reduces the fruit's visual appeal, market value and shelf life (Wang *et al*., 2023). Despite these patterns being recognized, the molecular mechanisms underlying apple senescence remain poorly understood. Clarifying these regulatory mechanisms is crucial for advancing high‐quality, efficient apple production.

Natural senescence marks the final stage of plant growth, driven by complex physiological and biochemical processes. It is primarily caused by an imbalance in reactive oxygen species (ROS) production and clearance within plant cells. Excessive ROS can cause oxidative damage to cells, ultimately leading to cellular dysfunction and senescence‐related processes (Mittler *et al*., 2022; Wang *et al*., 2024a, 2024b; Zhu *et al*., 2023). In *Arabidopsis*, ROS accumulation promotes leaf senescence (Yang *et al*., 2018), and this process is affected by the gene *AtWRKY75*, which inhibits CAT2 degradation, thereby modulating ROS levels *in vivo* (Guo *et al*., 2017). *OsLG3* inhibits ROS accumulation and delays leaf senescence in rice (Lim *et al*., 2024). Tulip *TgNAP* can regulate salicylic acid (SA) and ROS levels to promote petal senescence (Meng *et al*., 2022), and the rose PIF8‐BBX28 module can mediate petal senescence by affecting ROS homeostasis in mitochondria (Zhang *et al*., 2021). *Arabidopsis* CLE4 can act as a ‘brake signal’ to promote JuB1‐mediated ROS clearance and inhibit the senescence process of leaves (Zhang *et al*., 2023). In addition, ROS also functions as signalling molecules, activating senescence‐related genes like senescence‐associated genes (*SAGs*), polyamine oxidase genes (*PAOs*), clock‐controlled genes (*CCGs*), abscisic acid aldehyde oxidase genes (*AAOs*), lipoxygenase genes (*LOXs*), flavin adenine dinucleotide genes (*FADs*), superoxide dismutase genes (*SODs*), late embryogenesis abundant protein genes (*LEAs*), peroxidase genes (*PODs*), phenylalanine ammonia‐lyase genes (*PALs*), cinnamyl alcohol dehydrogenase genes (*CADs*), polyphenol oxidase genes (*PPOs*), and laccases genes (*LACs*). The activation of these genes results in senescence symptoms: chlorophyll degradation, decreased photosynthetic activity, yellowing leaves, dull fruit coloration, and the appearance of brown spots on the fruit peel (Chen *et al*., 2023; Wang *et al*., 2023; Zhang *et al*., 2018). However, previous research on the senescence mechanism has mainly focused on leaves and petals; less research has focused on natural fruit senescence. The senescence of fruits also directly affects the formation and maintenance of fruit quality, as well as market value and postharvest life (Giovannoni, 2001). In fruits such as tomato, kiwi, grape, peach, pear, loquat, and litchi, superoxide anions ($O_{2}^{-\cdot }$) and hydrogen peroxide (H_2_O_2_) levels often rise two‐ to threefold or more during ripening and senescence (Tian *et al*., 2013). However, the regulatory mechanism of ROS levels in fruit senescence is still not fully understood, so understanding this process more fully is essential for developing strategies to delay senescence and maintain fruit quality.

Plant maturation and senescence are regulated by numerous transcription factors that individually or cooperatively control specific downstream genes, such as MYB, WRKY, NAC, and ERF (Kuang *et al*., 2012; Shan *et al*., 2012; Xiao *et al*., 2013; Zhao *et al*., 2013). Recent studies have focused on WRKY transcription factors in plant senescence. For instance, in rice, OsWRKY42 suppresses *OsMT1d* expression, limiting ROS removal and accelerating leaf senescence (Han *et al*., 2014). The interaction of jasmonic acid (JA)‐induced protein ESR with AtWRKY53 reduces its DNA‐binding activity, leading to delayed senescence (Miao and Zentgraf, 2007). In *Arabidopsis*, various WRKY factors regulate senescence. For example, AtWRKY45 promotes natural senescence by modulating SAGs (Chen *et al*., 2017), AtWRKY57 inhibits senescence by repressing *SEN4/SAG12* (Jiang *et al*., 2017) and AtWRKY6 affects both senescence and pathogen defence through the senescence‐induced receptor kinase pathway. AtWRKY42 can regulate SA and ROS synthesis and positively regulate leaf senescence (Niu *et al*., 2020). In addition, previous studies have shown that in apple fruits under dark conditions, the MdWRKY31 positively regulates the expression of *MdLAC7* and promotes peel browning. The light response factor MdHY5 binds to the *MdWRKY31* and *MdLAC7* promoter, inhibits their activity and reduces browning incidence (Wang *et al*., 2023). Our study uncovered *MdWRKY70L* as a key modulator of apple fruit senescence through transcriptomic analysis. Molecular tests, including transient injection, stable overexpression and CRISPR/Cas9 knockout, demonstrated that *MdWRKY70L* promotes fruit senescence. These findings offer new insights into WRKY transcription factors' roles in apple fruit senescence, opening pathways for future research and potential interventions to manage fruit senescence.

Beyond transcriptional regulation, the mitogen‐activated protein kinase (MAPK) signalling cascade is critical for plant growth and development, with WRKY transcription factors as key downstream substrates (Sun and Zhang, 2021). For example, the overexpression of *AtWRKY53* promotes senescence, and MEKK1 phosphorylates WRKY53, enhancing its DNA‐binding ability. Moreover, WRKY53 can bind to its own promoter region, allowing it to be expressed not only during leaf senescence but throughout the plant senescence process (Miao *et al*., 2004). In our study, we also discovered that MdMPK6/02G interacts with MdWRKY70L, with phosphorylation modulating MdWRKY70L activity and enhancing its stability. This interaction affects ROS levels in fruits, ultimately regulating the fruit senescence process. These findings provide robust evidence for transcriptional and post‐translational regulation mechanisms related to fruit senescence and offer valuable insights for strategies aimed at maintaining postharvest fruit quality and extending storage time.

## Results

### Ultrastructure and ROS dynamics during apple fruit senescence

**Figure 1:**
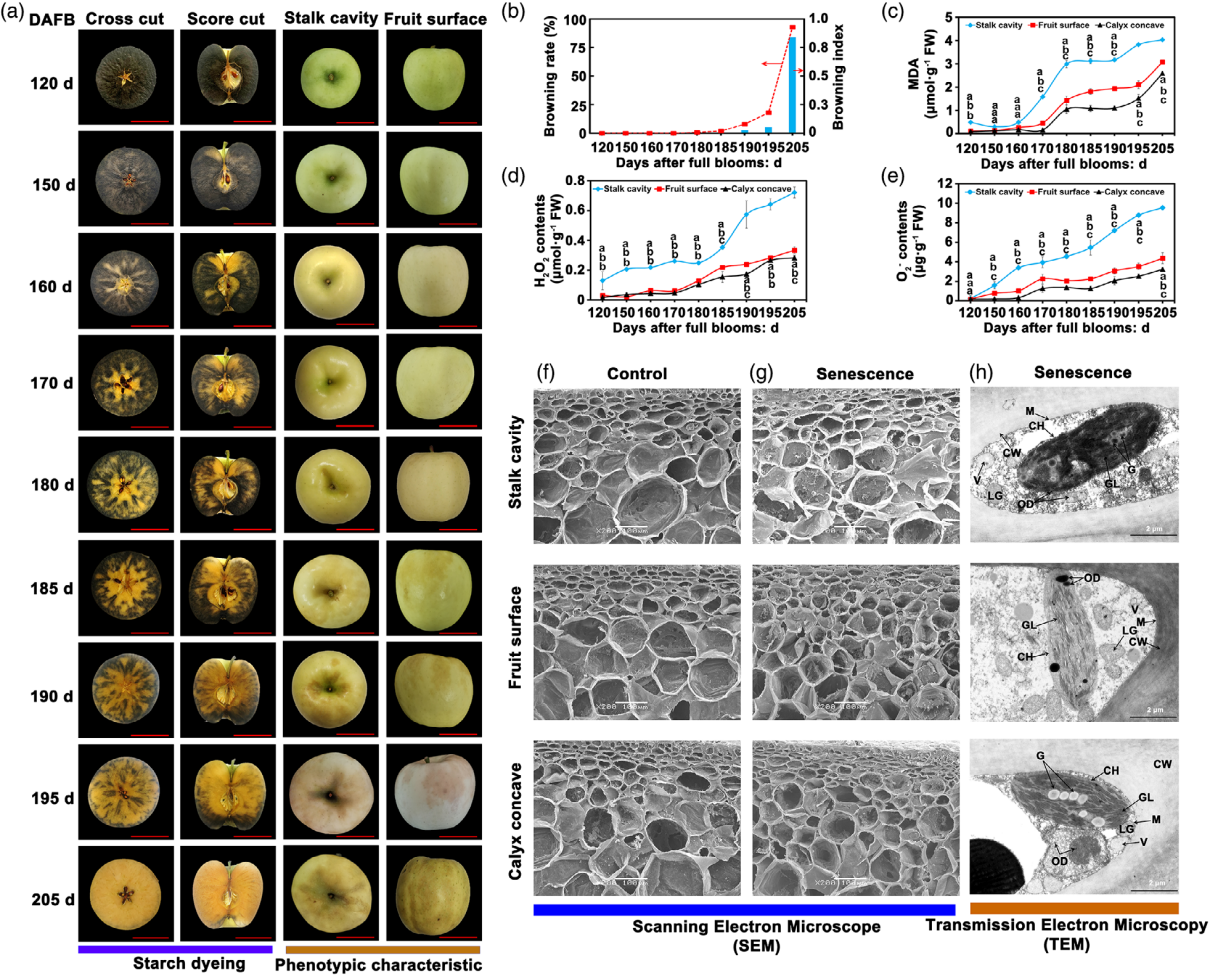
Changes in senescence characterization, ROS system, and ultrastructure in different parts of apple fruit during senescence. (a) Starch dyeing and phenotypic analysis of apples at different developmental stages. Digital images were extracted for comparison. Bars = 4 cm. (b) Analysis of the senescence browning index and rate of apples at different stages (*n* = 300 fruits). (c–e) Contents of MDA, $O_{2}^{-\cdot }$, and H_2_O_2_. The *x*‐axis indicates sampling time. Data are presented as mean ± SD with nine fruits per measurement. (f–h) Ultrastructure of the non‐senescent and senescent cell wall (CW), vacuole (V), chloroplast (CH), lipid globules (LG), mitochondria (M), osmiophilic droplets (OD), grain (G), and grana lamella (GL).

Starch staining effectively indicates fruit ripening and senescence; it was found that apple maturation follows a top‐to‐bottom gradient. Ripening begins in the stalk cavity, moves to the fruit surface, and finally reaches the calyx concave (Figure 1a). As maturation progresses, the stalk cavity initiates senescence, marked by increasing peel browning severity. The fruits begin browning at 180 days after full blooms, and by 205 days after full blooms, the browning rate reached 93% and the browning index was 0.84 (Figure 1a,b). Chlorophyll content also declines gradually across fruit regions, with the steepest reduction in the stalk cavity (Figure S1a). Analyses of antioxidant and ROS dynamics revealed a progressive decline in total phenols, flavonoids, flavanols, and overall antioxidant capacity during senescence, especially in the stalk cavity (Figure S1b–e). Meanwhile, levels of malondialdehyde (MDA), H_2_O_2_, and $O_{2}^{-\cdot }$ in the peel increased during fruit senescence, particularly in the stalk cavity (Figure 1c–e), where ROS‐scavenging enzyme activity was notably reduced. Compared with 120 days after full blooms of fruits in the stalk cavity, the activities of SOD, POD, and CAT in the stalk cavity of fruits at 205 days were significantly decreased by 65.4%, 82.7%, and 91.1%, respectively (Figure S1f–h). Ultrastructure examination showed that, in unripe fruits, peel cells in the stalk cavity, surface, and calyx concave exhibited a honeycomb‐like structure with smooth, intact cells (Figure 1f). However, with senescence onset, especially in the stalk cavity and surface, cells showed deformation, cell wall thickening, subepidermal cells sinking, and general tissue disorganization, losing the honeycomb pattern (Figure 1g). Moreover, starch particles were nearly absent in the cells at the browning sites, whereas the number of osmiophilic droplets increased, and chloroplast degradation was evident. By contrast, cells in the calyx concave, which exhibited no browning, retained visible starch particles, had fewer osmiophilic droplets, and displayed intact chloroplast structure, with minimal degradation of the thylakoid grana (Figure 1h). These findings indicated that apple fruit follows a pattern of longitudinal gradient maturation, beginning at the stalk cavity and extending towards the calyx concave. At full maturity, the fruit enters the senescence stage, starting from the stalk cavity. The decrease in antioxidant capacity and cell damage and a significant increase in ROS are the primary factors contributing to fruit senescence.

### MdWRKY70L as a key regulator of apple fruit senescence

**Figure 2:**
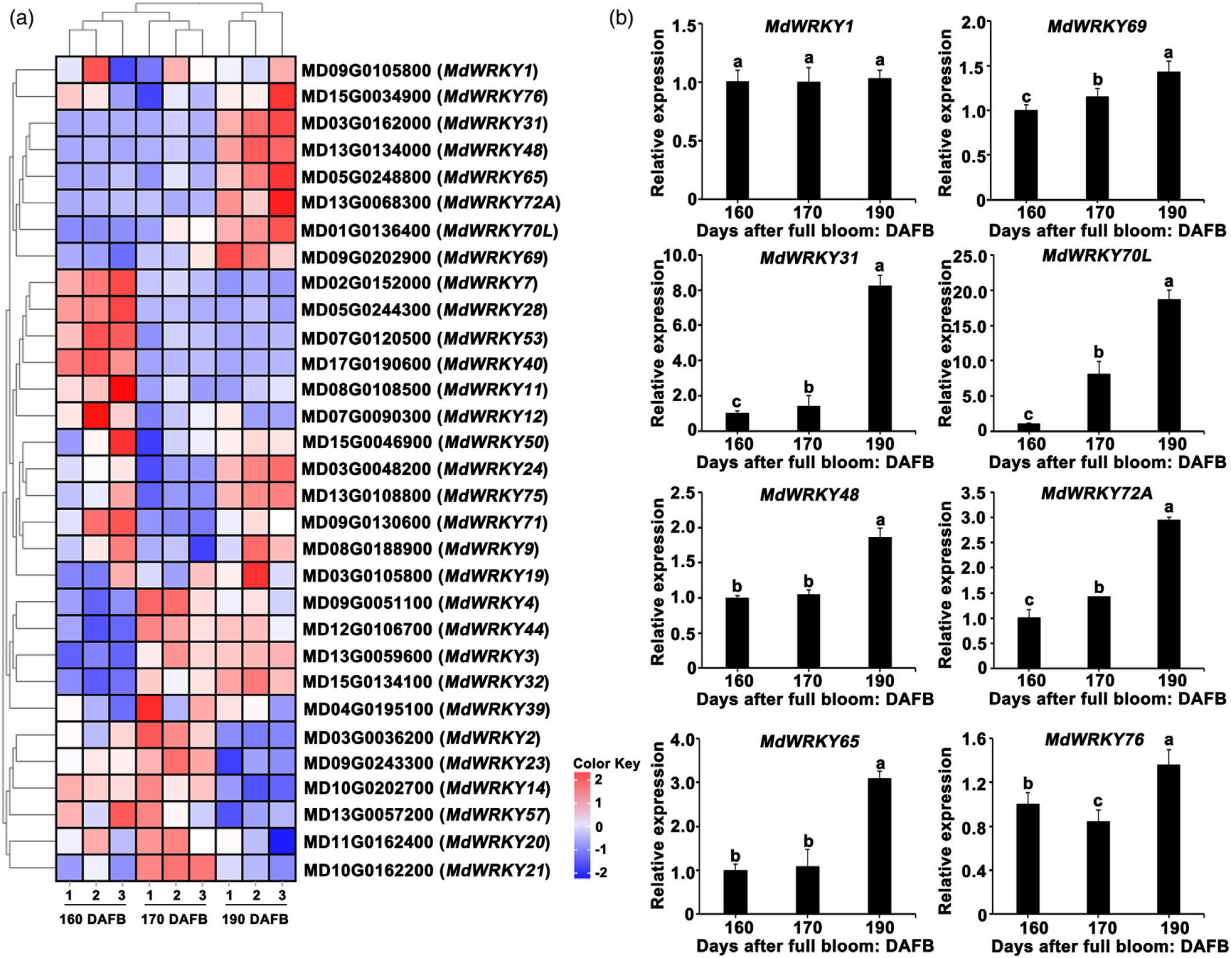
Screening of *MdWRKY70L* transcription factors. (a) Expression profiles of *MdWRKY* family genes. (b) Differentially expressed genes *MdWRKY1*/*3*/*31*/*24*/*48*/*65*/*69*/*70L*/*72A*/76 were identified. Data shown are means ± standard errors with different letters denoting *P* < 0.05 (Student's *t*‐test).

Research has shown that WRKY transcription factor family genes are integral to regulating senescence in crops (Zhou *et al*., 2011). To investigate WRKY genes linked to apple peel senescence, we performed clustering and enrichment analysis on WRKY family genes using transcriptome data across different developmental stages of fruits. In total, 31 WRKY family genes exhibited differential expression. Among these, the expression levels of *MdWRKY1* (MD09G0105800), *MdWRKY3* (MD13G0059600), *MdWRKY31* (MD03G0162000), *MdWRKY24* (MD03G0048200), *MdWRKY48* (MD13G0134000), *MdWRKY65* (MD05G0248800), *MdWRKY69* (MD09G0202900), *MdWRKY70L* (MD01G0136400), *MdWRKY72A* (MD13G0068300), *MdWRKY75* (MD13G0108800), and *MdWRKY76* (MD15G0034900) exhibited a gradually increasing expression as fruit senescence progressed (Figure 2a). Further reverse transcription quantitative polymerase chain reaction (RT‐qPCR) analysis confirmed that *MdWRKY70L* exhibited the highest differential expression among these genes (Figure 2b), and its expression was highest in the stalk cavity of the brown fruit (Figure S2), suggesting that the elevated *MdWRKY70L* expression is strongly associated with fruit senescence.

### MdWRKY70L promotes senescence in apple and ‘Orin’ calli

**Figure 3:**
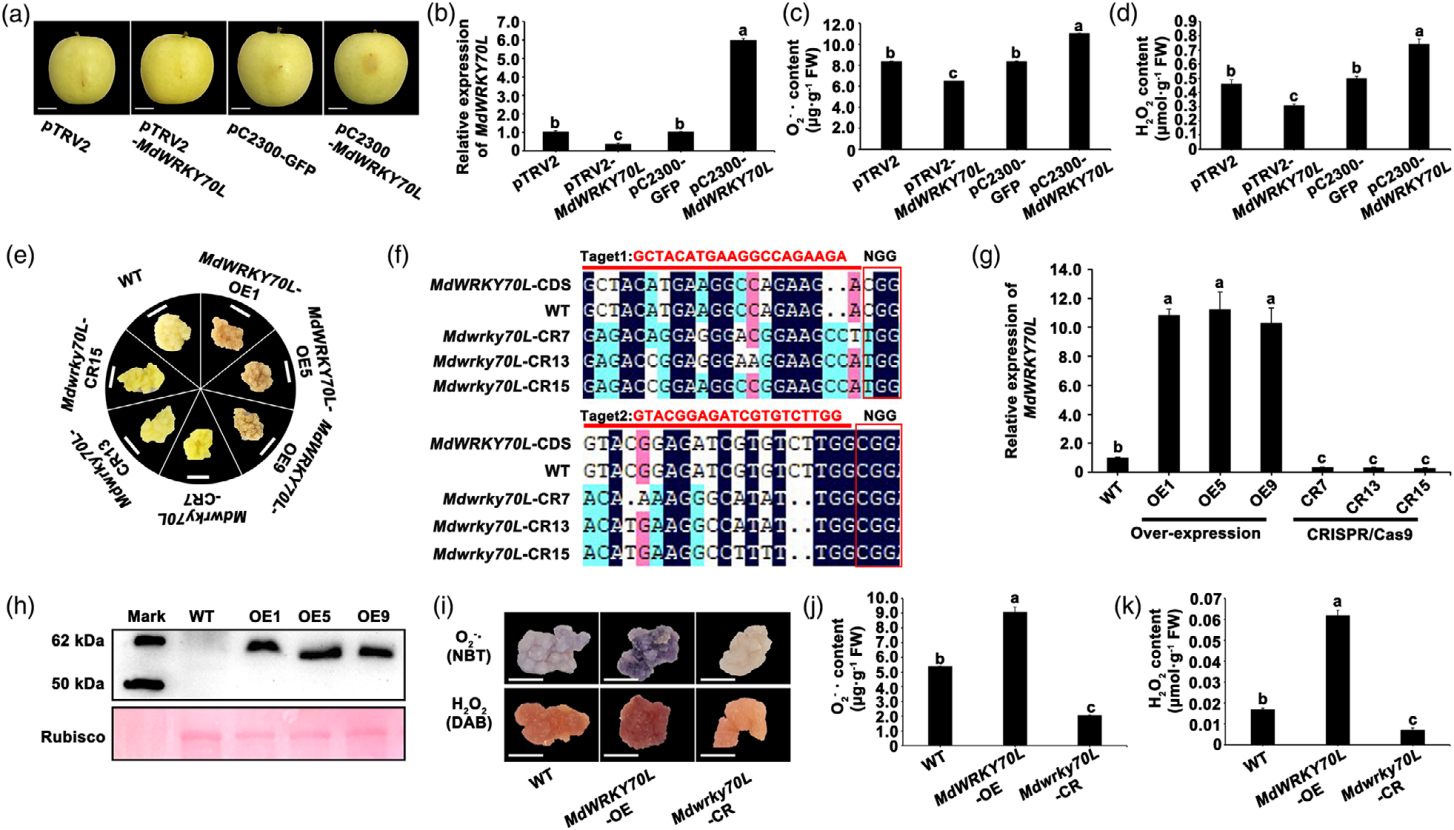
Functional validation of *MdWRKY70L* transcription factor. Effects of *MdWRKY70L* on apple phenotypes, with digital images for comparison. Bars = 2 cm. (b) *MdWRKY70L* expression and (c) $O_{2}^{-\cdot }$ and (d) H_2_O_2_ contents in apple peels post‐transient *MdWRKY70L* integration. Data shown are mean ± standard error with different letters denoting *P* < 0.05 (Student's *t‐*test). (e) Phenotype of *MdWRKY70L* overexpressing ‘Orin’ calli (OE‐*MdWRKY70L*‐1/5/9) and CRISPR/Cas9 knockdown calli (CR‐*MdWRKY70L*‐7/13/15). Bars = 1 cm. (f) Sequence verification of transgenic knockout materials. Sequences were aligned using DNAMAN. Before NGG was the target sequence. The dark region was the target sequence, and the other coloured region was the difference in sequence among the lines indicated. There was no difference between WT (wild‐type ‘Orin’) calli and target sequence, and multiple base mutations appeared in the knockdown calli (CR‐*MdWRKY70L*‐7/13/15). (g–h) *MdWRKY70L* RNA (g) and protein (h) levels in stable overexpressing lines. (i) NBT and DAB staining results of ‘Orin’ calli with *MdWRKY70L* stable overexpression and CRISPR/Cas9‐mediated knockout. Bars = 1 cm. (j) $O_{2}^{-\cdot }$ content. (k) H_2_O_2_ content. Data shown are mean ± standard error with different letters denoting *P* < 0.05 (Student's *t‐*test).

To assess the role of MdWRKY70L in apple fruit senescence, we constructed *MdWRKY70L* overexpression vectors (pCAMBIA2300‐*MdWRKY70L*), and silencing vectors (pTRV2‐*MdWRKY70L*), which were transiently transformed into apple peel tissue by using *Agrobacterium tumefaciens* as a mediator. Overexpression of *MdWRKY70L* significantly increased *MdWRKY70L* expression and accelerated senescence on the peel surface compared with the empty vector control (pCAMBIA2300‐GFP). By contrast, no senescence phenotype was observed following *MdWRKY70L* silencing (Figure 3a,b). ROS assays showed that in peel tissues overexpressing *MdWRKY70L*, the contents of $O_{2}^{-\cdot }$ and H_2_O_2_ increased by 24.5% and 32.4%, respectively, compared with the pCAMBIA2300‐GFP control. By contrast, in peel tissues with *MdWRKY70L* silencing, $O_{2}^{-\cdot }$ and H_2_O_2_ levels were significantly reduced by 21.7% and 32.6%, respectively (Figure 3c,d). We further confirmed these results in ‘Orin’ calli with stable overexpression and knockout of *MdWRKY70L* (Figure 3e–h). In the overexpressing calli, *MdWRKY70L* expression was significantly increased (Figure 3g), and $O_{2}^{-\cdot }$ and H_2_O_2_ levels rose by 0.7‐ and 2.6‐fold, respectively, compared to wild‐type (WT) calli, showing severe senescence (Figure 3i–k). In knockout calli, *MdWRKY70L* expression was nearly undetectable, with $O_{2}^{-\cdot }$ and H_2_O_2_ contents reduced by 61.8% and 58.8%, respectively, resulting in a youthful appearance with no senescence signs (Figure 3i–k). Subsequent activity measurements of antioxidant enzymes (SOD, POD, and CAT) showed that the enzyme activity in apple fruit and ‘Orin’ calli was significantly decreased regardless of whether *MdWRKY70L* was transiently or stably overexpressed. However, in *MdWRKY70L*‐silenced or knocked‐out materials, the enzyme activity was higher compared to both the control and overexpressed materials (Figure S3a–f). These findings demonstrate that *MdWRKY70L* is essential for driving the senescence process in both apple fruit and ‘Orin’ calli.

### MdWRKY70L regulates senescence‐related genes in apple fruits

**Figure 4:**
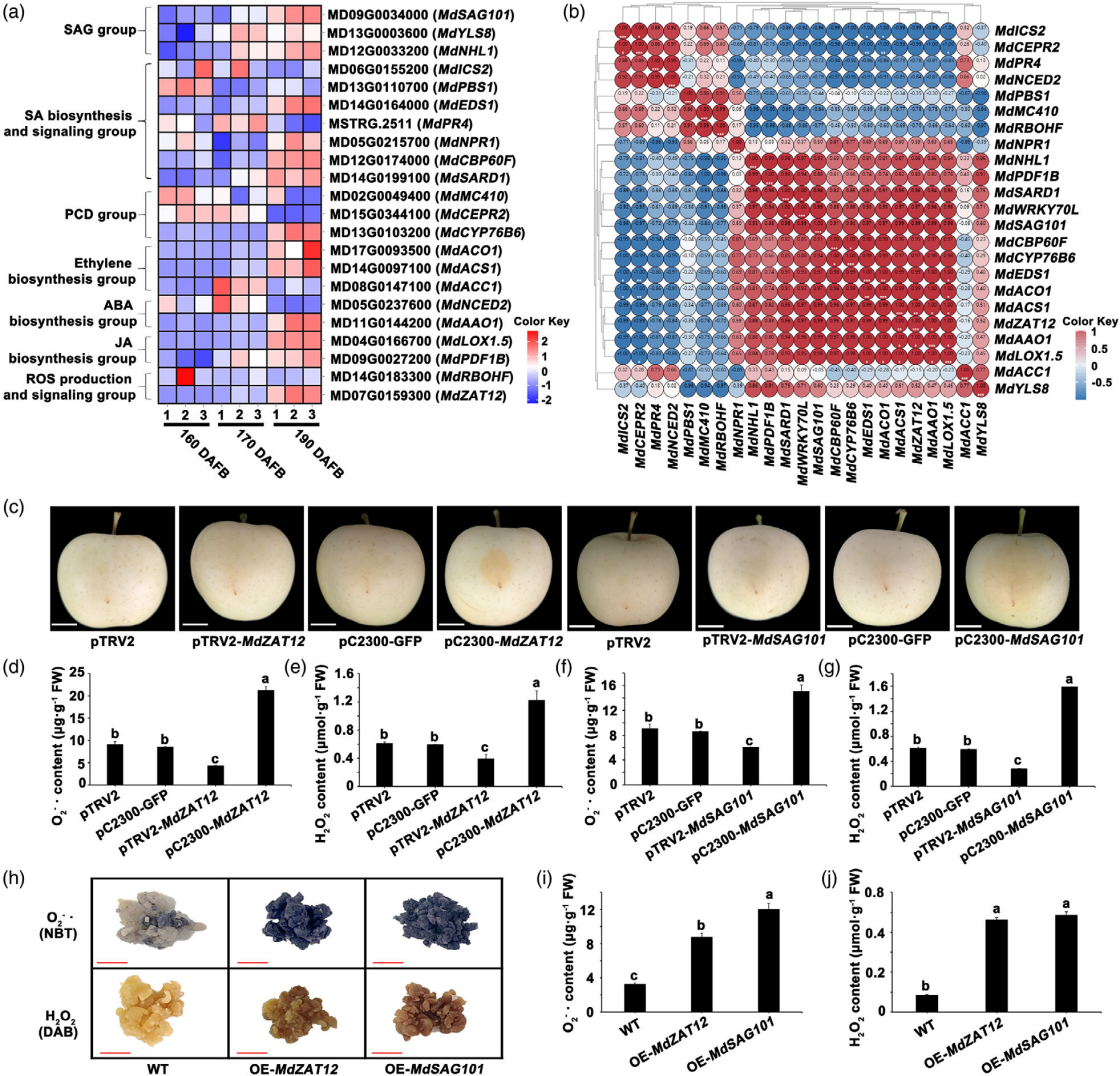
Screening and functional verification of senescence‐related genes. (a) Expression profiles of senescence‐related genes in apples at different developmental stages. (b) Correlation of *MdWRKY70L* with senescence‐related genes. (c) Phenotypes of apples after transient *MdSAG101* and *MdZAT12* infection. Digital images were isolated for comparison. Bars = 2 cm. (d–g) $O_{2}^{-\cdot }$ and H_2_O_2_ contents in apple after instant infection with *MdSAG101* and *MdZAT12*. Data shown are mean ± standard error with different letters denoting *P* < 0.05 (Student's *t‐*test). (h) Phenotype of ‘Orin’ calli stably overexpressing *MdSAG101* and *MdZAT12* after staining with NBT and DAB. Bars = 2 cm. (i) $O_{2}^{-\cdot }$ contents. (j) H_2_O_2_ contents.

To uncover the regulatory mechanism of MdWRKY70L in fruit senescence, we analysed DEGs from transcriptome data. This analysis revealed that SAGs, programmed cell death family genes and genes involved in salicylic acid, ethylene, abscisic acid, JA, and ROS biosynthesis were significantly enriched (Figure 4a). RT‐qPCR analysis further revealed that the levels of *MdSAG101* (MD09G0034000), *MdEDS1* (MD14G0164000), *MdCBP60F* (MD12G0174000), *MdCYP76B6* (MD13G0103200), *MdACO1* (MD17G0093500), *MdACS1* (MD14G0097100), *MdAAO1* (MD11G0144200), *MdLOX1.5* (MD04G0166700), and *MdZAT12* (MD07G0159300) increased as peel senescence progressed (Figure S4). Correlation analyses showed significant positive correlations between *MdWRKY70L* and both *MdSAG101* and *MdZAT12*, with correlation coefficients of 0.99 and 0.97, respectively (Figure 4b), suggesting that *MdWRKY70L* may accelerate peel senescence by regulating *MdSAG101* and *MdZAT12*, as their expression was augmented after transient *MdWRKY70L* injection and in stable *MdWRKY70L* transgenic ‘Orin’ calli. We found that the overexpression of *MdWRKY70L* promoted *MdSAG101* and *MdZAT12* expression, whereas silencing or knocking out *MdWRKY70L* significantly inhibited *MdSAG101* and *MdZAT12* expression. Notably, *MdSAG101* and *MdZAT12* exhibited the largest difference in variation (Figure S5a,b).

To further validate the roles of *MdSAG101* and *MdZAT12* in fruit senescence, we transformed the pTRV2‐*MdSAG101*/pTRV2‐*MdZAT12* gene silencing vectors and the pCAMBIA2300‐*MdSAG101*/pCAMBIA2300‐*MdZAT12* overexpression vectors into apple peel tissue, using *Agrobacterium* as the mediator. Compared to controls (pCAMBIA2300‐GFP and pTRV2), overexpressing *MdSAG101* and *MdZAT12* significantly augmented $O_{2}^{-\cdot }$ and H_2_O_2_ levels in the peel, whereas silencing these genes reduced $O_{2}^{-\cdot }$ and H_2_O_2_ contents (Figure 4c–g). In addition, we successfully obtained ‘Orin’ calli with stable overexpression of *MdSAG101* and *MdZAT12* genes (Figure S6a–f). In these overexpressed ‘Orin’ calli, $O_{2}^{-\cdot }$ and H_2_O_2_ levels were also significantly higher than that in the WT (Figure 4h–j), while antioxidant activity was significantly lower than that in the WT (Figure S7). These observations suggested that MdWRKY70L may regulate *MdSAG101* and *MdZAT12* expression, thereby mediating ROS accumulation in the peel and accelerating the fruit senescence process.

### MdWRKY70L positively regulates *MdSAG101* / *MdZAT12* expression to promote apple fruit senescence

**Figure 5:**
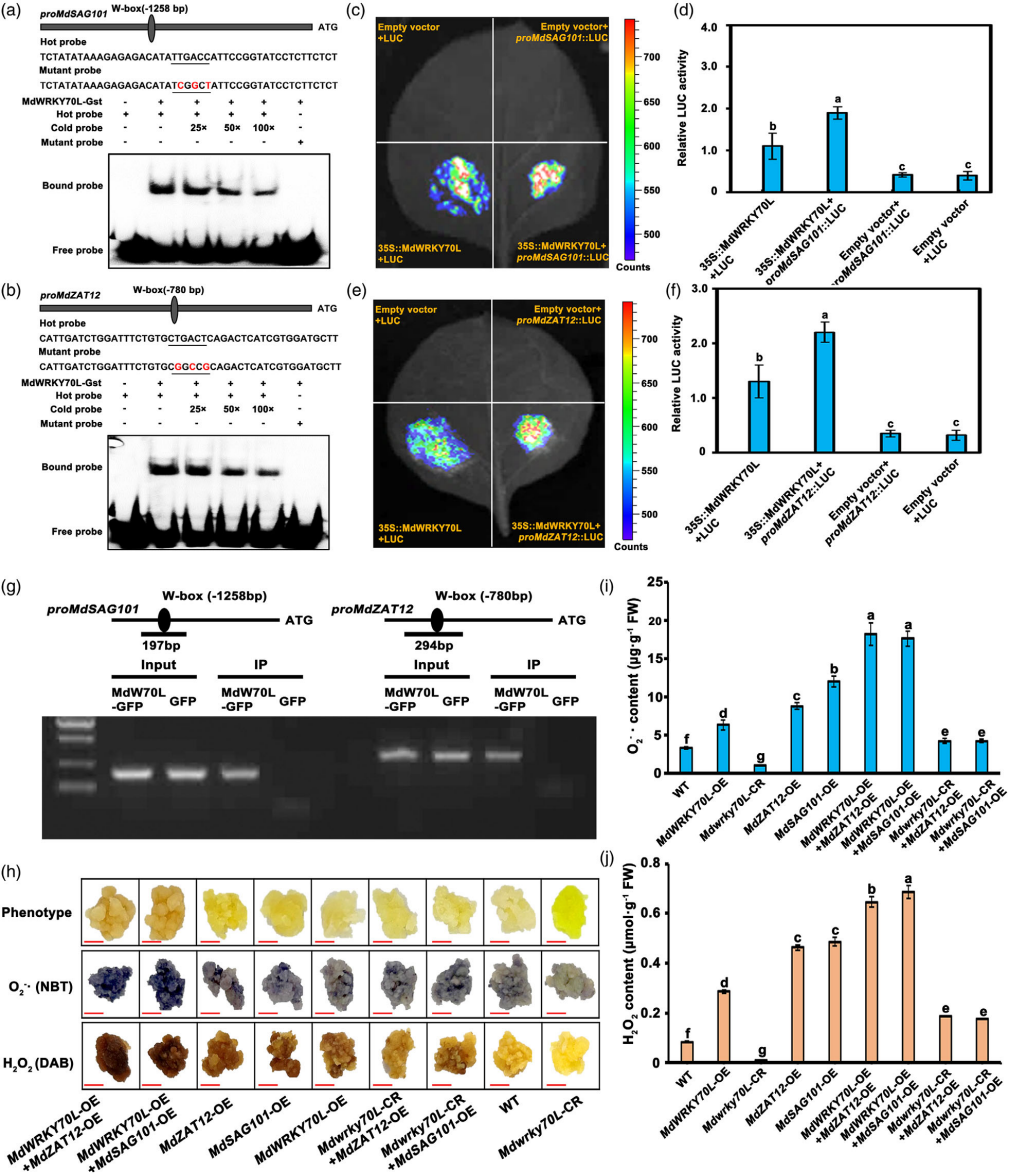
MdWRKY70L binding to *MdSAG101*/*MdZAT12*'s promoter. (a, b) Electrophoretic mobility shift assay using biotin‐labelled and unlabelled promoter probes specific to *MdSAG101*/*MdZAT12*'s W‐box motifs and a mutated probe indicating MdWRKY70*L* binding to *MdSAG101*/*MdZAT12* promoter. Cold probes were provided incrementally (25×, 50× and 100×). The ‘+’ and ‘−’ symbols denote the inclusion and exclusion of each probe or protein, respectively. (c–f) Luciferase analysis uncovering *in vivo* MdWRKY70L binding to the *MdSAG101*/*MdZAT12* promoters in agroinfiltrated *Nicotiana benthamiana* leaves on day 3. (g) Binding of MdWRKY70L to the *MdSAG101*/*MdZAT12* promoters *in vivo* in ChIP‐PCR assay. (h) Calli phenotype after NBT staining for $O_{2}^{-\cdot }$ with darker colours representing higher contents and DAB for H_2_O_2_ with browner colours representing higher contents. Bars = 1 cm. (i) $O_{2}^{-\cdot }$ contents. (j) H_2_O_2_ contents. Data shown are means ± standard errors with different letters denoting *P* < 0.05 (Student's *t‐*test).

To investigate how MdWRKY70L promotes senescence, we analysed the promoters of *MdSAG101* and *MdZAT12* and identified W‐box motifs (WRKY‐binding sites, TTGACC/CTGACT). The electrophoretic mobility shift assay (EMSA) was used to verify whether MdWRKY70L binds to these sites using probes (hot, cold, and mutant probes) specifically designed for these sites. When the purified MdWRKY70L‐GST protein was co‐incubated with the hot probe, MdWRKY70L was found to bind to the W‐box probe on the *MdSAG101* and *MdZAT12* promoters, while the cold probes weakened the DNA‐binding ability. The mutant probes had no binding ability (Figure 5a,b). These observations suggested that MdWRKY70L physically binds to the W‐box on the *MdSAG101* and *MdZAT12* promoters. We further used the luciferase (LUC) assay to analyse whether MdWRKY70L transcriptionally activates *MdSAG101* and *MdZAT12 in vivo*. It was found that MdWRKY70L binding to these promoters activates their expression in plant cells (Figure 5c–f). In addition, the ChIP‐PCR assay results also showed the interaction between them; the *MdSAG101* and *MdZAT12* promoter fragments containing the W‐box site were substantially enriched in the MdWRKY70L‐GFP ‘Orin’ calli (relative to the control level; Figure 5g). These results indicated that MdWRKY70L binds to their promoters and transcriptionally activates *MdSAG101* and *MdZAT12 in vivo*.

To explore whether *MdWRKY70L* promotes fruit senescence by modulating *MdSAG101* and *MdZAT12*, we separately transformed *MdSAG101* and *MdZAT12* into *MdWRKY70L* overexpression and knockout ‘Orin’ calli. The results showed that in *MdWRKY70L* knockout calli, the overexpression of *MdSAG101* and *MdZAT12* induced senescence phenotypes, and the $O_{2}^{-\cdot }$ and H_2_O_2_ contents significantly increased compared with the WT (Figure 5h–j). By contrast, in *MdWRKY70L* overexpression calli, the stable transformation of *MdSAG101* and *MdZAT12* intensified senescence phenotypes, with markedly higher $O_{2}^{-\cdot }$ and H_2_O_2_ levels than the WT (Figure 5h–j). However, the activity of antioxidant enzymes in this material is significantly opposite to the accumulation trend of $O_{2}^{-\cdot }$ and H_2_O_2_ contents (Figure S8). These observations demonstrated that MdWRKY70L acts in conjunction with *MdSAG101* and *MdZAT12*, both *in vivo* and *in vitro*, and can jointly promote ROS accumulation, thus accelerating the apple fruit senescence.

### MdMPK6/02G interacts with MdWRKY70L and enhances its protein stability

**Figure 6:**
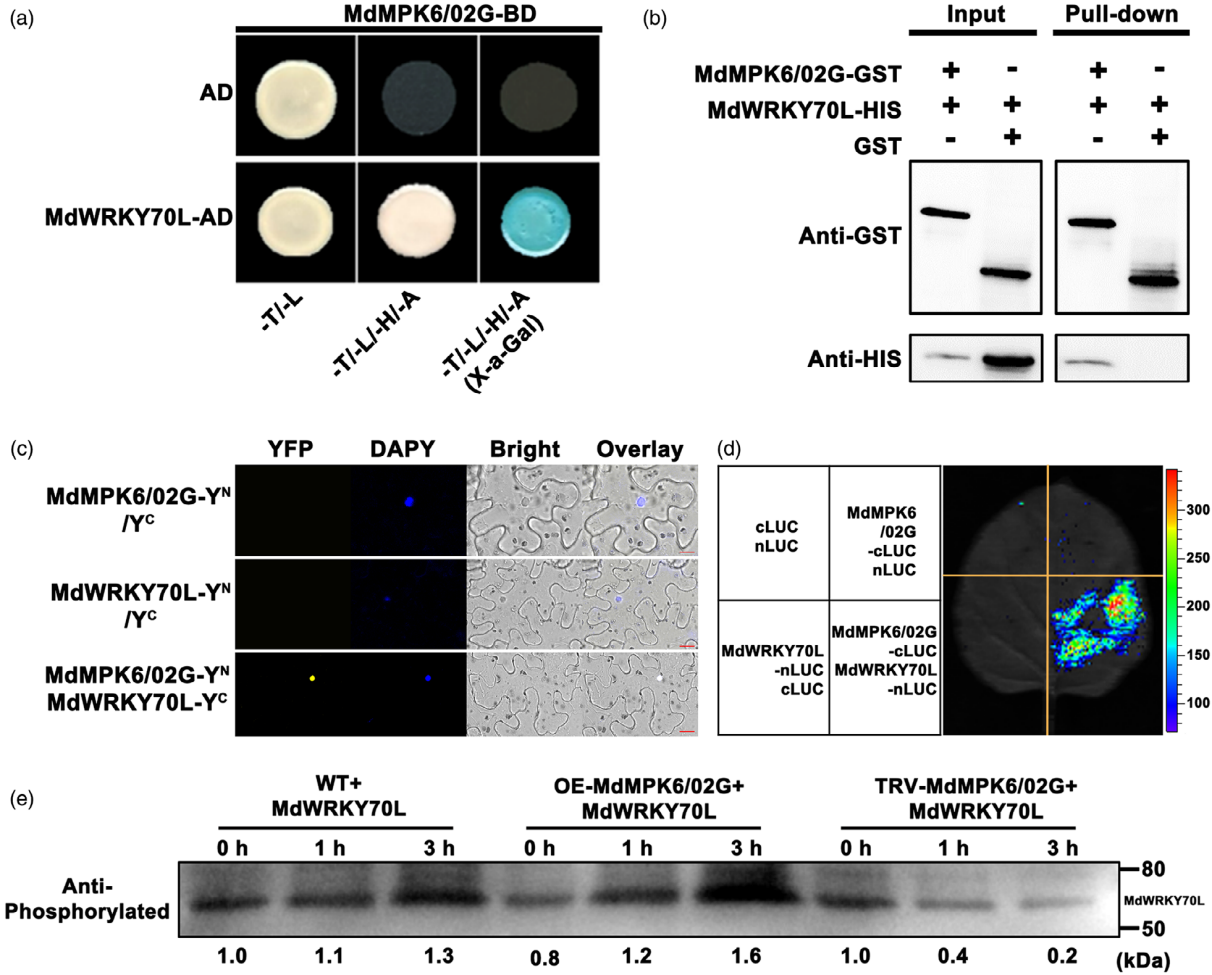
*In vivo and in vitro* interactions between MdMPK6/02G and MdWRKY70L enhance MdWRKY70L stability. (a) Y2H assays. MdMPK6/02G interacted with MdWRKY70L. The empty pGADT7 vector (AD) served as a negative control. Blue plaque indicates interactions between MdMPK6/02G and MdWRKY70L. (b) Pull‐down assay. ‘+’ and ‘−’ indicate the presence and absence, respectively, of the indicated protein. (c) MdMPK6/02G interacted with MdWRKY70L in bimolecular fluorescence complementation (BIFC) assays. (d) Luciferase complementation imaging (LCI) assays showed that MdMPK6/02G interacted with MdWRKY70L. (e) Verification of protein phosphorylation stability *in vivo*. The number below the protein band indicates the relative abundance of the protein.

Protein modification, such as phosphorylation, is essential in regulating protein functions, with WRKY transcription factors often undergoing phosphorylation to facilitate plant growth and development. Using LC–MS/MS analysis on proteins extracted from *MdWRKY70L*‐GFP transgenic ‘Orin’ calli, we unveiled phosphorylated peptides in *MdWRKY70L*‐GFP samples, confirming that MdWRKY70L undergoes phosphorylation (Figure S9 and Table S1). To further explore this, we performed yeast two‐hybrid (Y2H) screening and observed associations between MdMPK6/02G and MdWRKY70L (Table S2). Specifically, yeast cells co‐expressing *MdWRKY70L*‐PGAD and *MdMPK6/02G*‐PGBK exhibited regular growth on the selective medium (−T/−L/−H/−A) and blue coloration, indicating interaction. Additional pull‐down, bimolecular fluorescence complementation (BiFC) and luciferase complementation imaging (LCI) assays further validated this protein–protein interaction (Figure 6a–d). Subsequently, *MdMPK6/02G*‐flag and *MdMPK6/02G*‐TRV2 vectors were transformed into ‘Orin’ calli, total protein was extracted, and purified MdWRKY70L‐GST protein was co‐incubated at 30 °C for 0, 1 and 3 h. Overexpression of MdMPK6/02G calli protein enhanced the stability of MdWRKY70L‐GST protein over time compared with *MdMPK6/02G*‐TRV2 and WT calli proteins (Figure 6e). These results indicated that MdMPK6/02G interacts with MdWRKY70L and enhances its protein stability.

### MdMPK6/02G promotes fruit senescence by phosphorylating MdWRKY70L at Ser199

**Figure 7:**
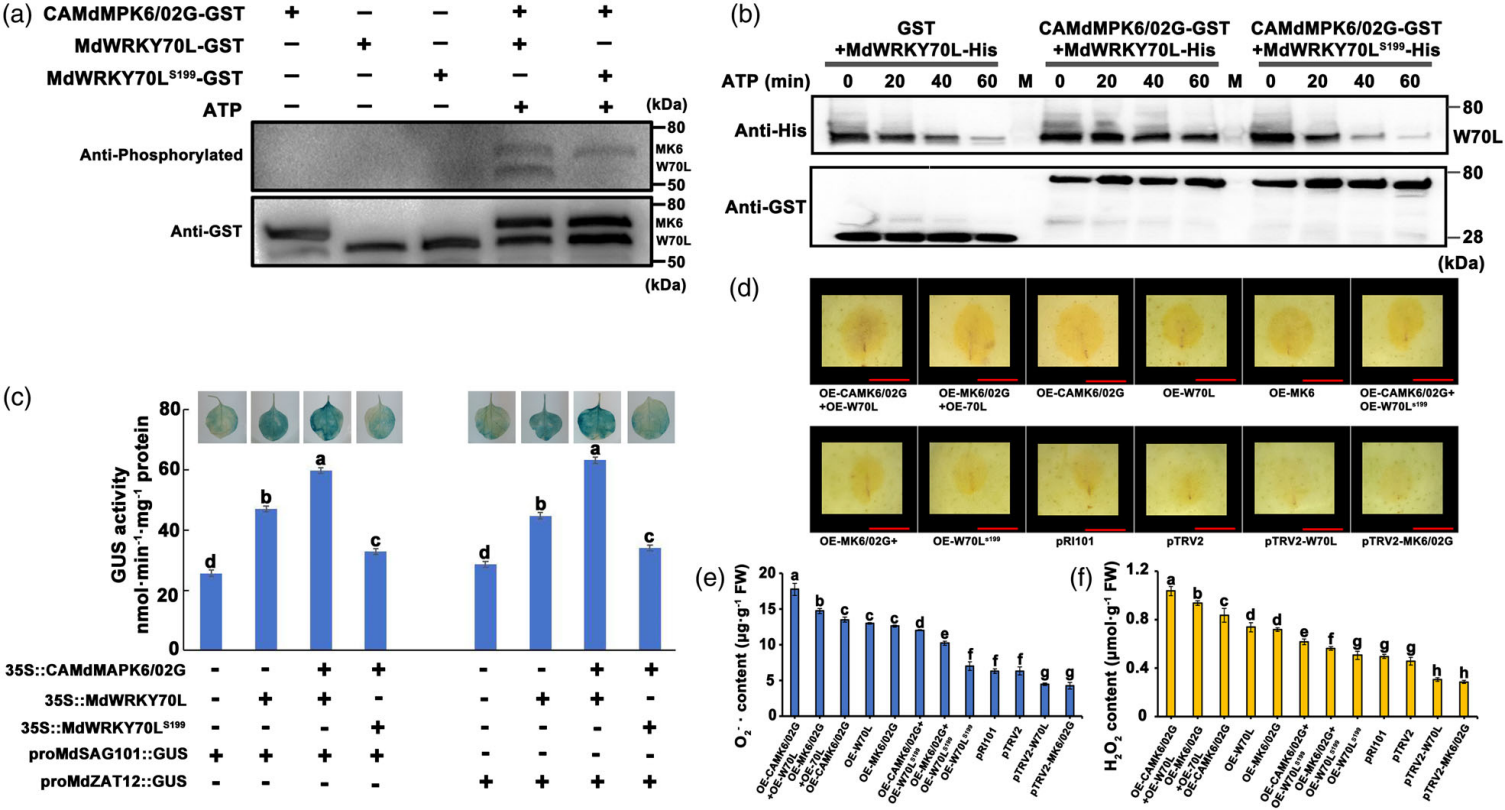
MdWRKY70L phosphorylation at Ser199 by MdMPK6/02G promotes fruit senescence. (a) Recombinant CAMdMPK6/02G‐GST, MdWRKY70L‐GST and MdWRKY70L^S199^‐GST (a mutation with loss of the phosphorylation site) were isolated after expression in BL21 (DE3) and used for phosphorylation assays *in vitro*. ‘+’ and ‘−’ indicate the presence and absence of the indicated protein, respectively. MK6 and W70L represent phosphorylated protein bands of MdMPK6/02G and MdWRKY70L, while MK6‐GST and W70L‐GST bands represent proteins added to the reaction. (b) Cell‐free protein degradation experiments showed that CAMdMPK6/02G‐GST inhibited the degradation of MdWRKY70L‐His protein. GST protein was used as a control, and equally purified CAMdMPK6/02G‐GST was incubated with recombinant MdWRKY70L^S199^‐His protein in the presence of ATP. The degree of protein degradation was detected by anti‐GST and anti‐His antibody after incubation for 0, 20, 40, and 60 min. The experiments were repeated independently at least three times, with similar results. (c) The GUS staining phenotype and GUS activity analysis in tobacco leaves. (d) Transient transgene integration verification for fruit senescence induced by phosphorylation of MdWRKY70L at Ser199 by MdMPK6/02G. Scale bar = 2 cm. Apple images were digitally processed for comparison. (e, f) $O_{2}^{-\cdot }$ and H_2_O_2_ contents in apples after instant infection with *MdMPK6/02G* and *MdWRKY70L*. Different lowercase letters indicate significant differences at *P <* 0.05 (Student's *t*‐test).

We identified phosphorylation at the Ser199 site of MdWRKY70L through immunoprecipitation and mass spectrometry (IP/MS) (Table S1). To verify the role of MdMPK6/02G in phosphorylating MdWRKY70L, we obtained active CAMdMPK6/02G‐GST protein and a point mutant version of MdWRKY70L with a Ser199 mutation (MdWRKY70L^S199^‐GST) for *in vitro* analysis. In an *in vitro* phosphorylation experiment using kinase buffer, we found that CAMdMPK6/02G could phosphorylate MdWRKY70L but was unable to phosphorylate MdWRKY70L^S199^, indicating that CAMdMPK6/02G regulates MdWRKY70L activity by phosphorylating it at the Ser199 site (Figure 7a). Further phosphorylation and degradation tests *in vitro* showed that, compared with GST protein, when CAMdMPK6/02G‐GST protein was co‐incubated with MdWRKY70L‐His protein, the degradation rate of MdWRKY70L‐His could be reduced, but CAMdMPK6/02G‐GST protein could not prevent the degradation of MdWRKY70L^S199^‐His protein (Figure 7b). This is consistent with the results of phosphorylation and degradation tests *in vivo* (Figure 6e).

Next, the GUS activity analysis showed that MdWRKY70L could promote the GUS activity of the promoters of *MdSAG101* and *MdZAT12*, and the addition of 35S::CAMAPK06/02G further enhanced their GUS activity. However, co‐injection of 35S::CAMAPK06/02G and 35S::MdWRKY70L^S199^ did not induce more GUS activity (Figure 7c). In addition, we conducted transient transformation experiments, overexpressing *MdWRKY70L*‐GFP, *MdWRKY70L* ^S199^‐GFP, *CAMdMPK6/02G*‐flag, *CAMdMPK6/02G*‐flag + *MdWRKY70L*‐GFP, and *CAMdMPK6/02G*‐flag + *MdWRKY70L* ^S199^‐GFP in the apple peels. The results showed that co‐transfection of *MdMPK6/02G*‐flag with *MdWRKY70L*‐GFP in the apple peel led to severe senescence phenotypes (Figure 7d and Figure S10), with a significant increase in $O_{2}^{-\cdot }$ and H_2_O_2_ levels (Figure 7e,f) and with a significant decrease in the antioxidant oxidase activity (Figure S11a–c). However, when the Ser199 site of *MdWRKY70L* was mutated, co‐transfection of *MdMPK6/02G*‐flag with *MdWRKY70L* ^S199^‐GFP alleviated the symptoms of peel senescence (Figure 7d and Figure S10). Moreover, the $O_{2}^{-\cdot }$ and H_2_O_2_ levels were significantly decreased (Figure 7e,f), and the antioxidant oxidase activity was significantly increased (Figure S11a–c). These effects were even more pronounced when *CAMdMPK6/02G*‐flag was expressed. In conclusion, MdMPK6/02G phosphorylates MdWRKY70L at the Ser199 site, thereby promoting senescence in apple peel.

## Discussion

Fruit growth and development can proceed through five stages: cell differentiation, cell expansion, fruit development, ripening and senescence, with natural senescence being the final stage. This stage is crucial as it directly affects fruit quality, market value and shelf life (Giovannoni, 2001). Fruit senescence is a complex, highly regulated physiological and biochemical process, which is tightly regulated and influenced by ROS accumulation (Buchanan‐Wollaston *et al*., 2005; Zhang et al., 2018; Zhu *et al*., 2018; Lokdarshi et al., 2020). As senescence progresses, physiological functions decline, cell damage occurs and pulp browning and decreased resistance to pathogens make the fruits more susceptible to spoilage, thus significantly shortening postharvest life and preservation time (Tian *et al*., 2013; Wang *et al*., 2004; Zhang et al., 2022; Wang *et al*., 2023). Previous studies have shown that under natural growth conditions, plant adaptations minimize the damage that could be induced by ROS. However, oxygen toxicity appears when ROS production exceeds the quenching capacity of the protective systems due to stress conditions (Mittler *et al*., 2017, 2022; Zhang *et al*., 2024a, 2024b). When bananas were exposed to low temperatures, the expression of PPO genes was up‐regulated by >100‐fold, leading to a ROS surge and subsequent peel browning (Zhu *et al*., 2020). When apples were in a high‐temperature environment, ROS accumulation was excessive, resulting in increased cell membrane permeability, breaking the partition of polyphenols and PPO and accelerating the senescence (browning) performance of the peel (Wang *et al*., 2024a, 2024b). In our study, ROS levels in various senescent fruit regions showed a progressive increase in $O_{2}^{-\cdot }$ and H_2_O_2_. Meanwhile, antioxidant enzyme activities and antioxidant compound levels declined, confirming that ROS accumulation is a major factor mediating fruit senescence. While these findings align with earlier research, most studies on plant senescence mechanisms have focused on leaves. Further explorations are needed to clarify the specific dynamics of ROS changes during fruit senescence.

WRKY transcription factors are essential in plants, where they regulate gene expression by binding to W‐box elements in promoter regions. These transcription factors function as either activators or repressors, influencing a range of processes, such as growth, responses to biotic and abiotic stresses and hormone signalling (Wang *et al*., 2023). In plant senescence, the tobacco transcription factor *NtWRKY70b* facilitates leaf senescence by inducing ROS accumulation and impairing hydrogen sulphide biosynthesis (Ahmad *et al*., 2024; Zhang *et al*., 2024a, 2024b). In the apple, MdVQ10 interacted with *MdWRKY75* to enhance *MdWRKY75*‐activated transcription of *MdSAG12*/*18*, thereby promoting plant senescence (Zhang *et al*., 2023). In fruit senescence, WRKY transcription factors often exhibit significant expression changes. For instance, in banana fruits, *MaWRKY31* activates the promoter activity of ethylene synthesis genes *MaACS1* and *MaACO1*, which may enhance ethylene synthesis and accelerate fruit senescence (Xiao *et al*., 2013). Similarly, in tomato fruits, several *SlWRKY* genes are up‐regulated during fruit maturation and contribute to post‐ripening regulation by controlling ethylene synthesis, pigment accumulation, fruit softening, and other related processes (Huang *et al*., 2022). Beyond ethylene synthesis, WRKY factors can directly target senescence‐associated genes, such as MaWRKY31's activation of *MaSAG1* in banana (Xiao *et al*., 2013), and modulate ROS levels, thereby mediating fruit senescence (Chen *et al*., 2017). In this study, MdWRKY70L was observed to bind to W‐box motifs in the promoters of *MdSAG101* and *MdZAT12*, actively regulating their expression, thereby affecting ROS levels and promoting fruit senescence. These results deepen our understanding of the transcriptional regulation pathways that control fruit senescence.

**Figure 8:**
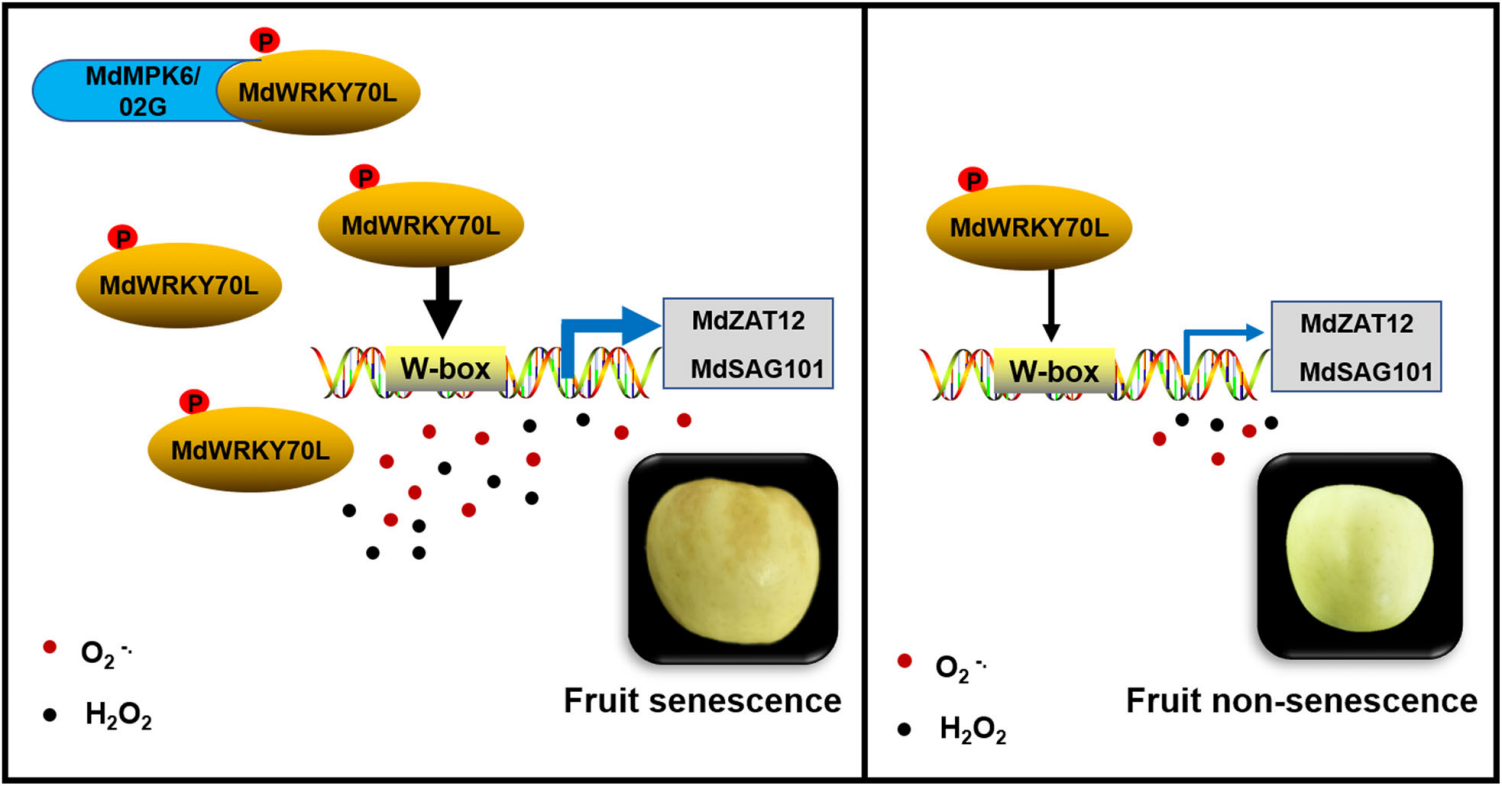
Proposed model for apple fruit senescence regulated by MdMPK6/02G‐mediated MdWRKY70L phosphorylation and ROS accumulation. *MdWRKY70L* TF is a key candidate gene that regulates apple fruit senescence. MdWRKY70L could interact with *MdSAG101* and *MdZAT12* both *in vivo* and *in vitro*, thereby mediating ROS production in the peel and accelerating the fruit senescence process. In addition, MdMPK6/02G phosphorylates and stabilizes MdWRKY70L, further promoting the senescence phenotype in apples.

WRKY transcription factor activity is primarily modulated through MAPK‐mediated phosphorylation. In *Arabidopsis*, AtWRKY33 phosphorylation by MPK3/MPK6 regulates plant antitoxin biosynthesis (Mao *et al*., 2011). Similarly, the absence of an MPK3/MPK6 phosphorylation site affects WRKY34 function *in vivo* (Guan *et al*., 2014). *OsWRKY53* negatively modulates MPK3/MPK6 to activate early plant defence responses (Hu *et al*., 2015) and interacts with the OsMAPKK4*–*OsMAPK6 cascade to influence brassinolide signalling (Tian *et al*., 2017). In addition, MPK1 phosphorylation of WRKY53 in *Arabidopsis* enhances its DNA‐binding ability, accelerating the leaf senescence process (Li *et al*., 2020). Our study unveiled that MdMPK6/02G phosphorylates MdWRKY70L at Ser199 to enhance its stability. This modification further promotes the regulation of the downstream senescence‐related genes *MdSAG101* and *MdZAT12*, leading to increased ROS accumulation and ultimately causing fruit senescence and browning (Figure 8). These findings offer promising potential for molecular‐assisted breeding to delay fruit senescence and preserve fruit quality.

## Materials and methods

### Plant materials and treatments

This study used 6‐year‐old ‘Ruixue’ apple trees and ‘Orin’ calli as test materials. The experiment took place from June to November 2022 at the Bai Shui Apple Experimental Station (35°02′N, 109°06′E, 908 m altitude) of Northwest A&F University. The site, located in a moderate monsoon climate with continental features, experiences a mean annual rainfall of 578 mm and a daily mean temperature of 11.4 °C.

To prepare samples, fruits were routinely bagged 55 days after full bloom (DAFB; June 15). Sampling began at 160 DAFB and continued at 10‐day intervals across four sampling points. At each time, 30 fruits of similar size, maturity, and without mechanical damage were selected from each group. The peel was carefully removed using a sterile scalpel, rapidly frozen in liquid nitrogen, and kept at −80 °C for later experiments.

For genetic transformation, ‘Orin’ calli were cultured on MS medium supplemented with 1.5 mg L^−1^ 2,4‐dichlorophenoxyacetic acid (2,4‐D) and 0.4 mg L^−1^ 6‐benzylaminopurine (6BA) in the dark at 25 °C and refreshed every 20 days. Meanwhile, *Nicotiana benthamiana* was cultivated under a 16 h/8 h light/dark cycle at 25 °C and 70% ± 5% relative humidity in a light incubator.

### Browning rate and index

The browning rate and index were assessed using a slightly modified method from Wang *et al*. (2023). The browning rate is the percentage of browned fruits in a sample of 300 randomly selected fruits. Browning severity (S) was rated on a 0–3 scale with 0, 1, 2, and 3 for no, mild (<1/3 of the fruit's surface), moderate (between 1/3 and 2/3), and severe (>2/3) browning, respectively. The browning index was determined as ∑ [(browning scale) × (fruit count at that scale)]/(3 × total fruit count) × 100.

### Antioxidant capacity

Total antioxidant activity and components were measured using a modified method based on Wang *et al*. (2023). 0.5 g of fresh peel samples were prepared as a fine powder and mixed with 1.5 mL of a 7:3 (v:v) ethanol–acetone solution. The mixture was kept at 37 °C for 1 h and spun for 10 min at 15 000 ***g*** and 20 °C. The resulting supernatant was instantly placed at −20 °C for subsequent antioxidant capacity analysis. The experiments were conducted in triplicate, with three biological replicates for accuracy.

### Histological staining for ROS detection

$O_{2}^{-\cdot }$ and H_2_O_2_ levels were detected as previously described, with slight modifications (Zhang *et al*., 2024a, 2024b). Briefly, the ‘Orin’ calli were immersed in 1 mg/mL DAB staining solution for H_2_O_2_ or 1 mg/mL NBT staining solution for $O_{2}^{-\cdot }$. The calli were incubated under shaking (20 rpm) at 25 °C overnight in the dark. After incubation, the calli were preserved in a solution of ethanol and glycerol (v:v = 4:1) until imaging was performed.

### Microstructure of the peel cells

For microstructural analysis, peel tissues (1.0 × 2.0 × 5.0 mm) were cut with a scalpel and rapidly fixed in 4% glutaraldehyde (v:v). The samples were vacuumed to ensure complete immersion in the fixative and left overnight. Following this, the samples were rinsed with phosphate buffer (PBS; 0.1 mol/L, pH 6.8) and fixed for 2 h in 1% osmium tetroxide. After five 5‐min PBS washes, the samples were dehydrated using a graded ethanol series (30%, 50%, 70%, 80%, and 90%), with each concentration applied for 10 min, followed by three 10‐min washes with 100% ethanol and embedding overnight in epoxy propane and SPI‐81 medium. Ultrathin sections (90 nm) were cut with a Leica EM UC7 ultramicrotome (Leica, Germany), stained with uranyl acetate and lead citrate and examined under a Hitachi HT7700 microscope (Hitachi, Japan) (Wang *et al*., 2023).

### RNA isolation and quantification

RNA was isolated with TRIzol (Invitrogen, Carlsbad, CA), and its integrity was evaluated using Agilent 2100 Bioanalyzers (Agilent Technologies, Palo Alto, CA) and agarose gel electrophoresis. Gene levels were analysed by RT‐qPCR with three biological replicates using SYBR Green Master Mix (SYBR Premix EX TaqTM, Dalian, China) on an ABI7500 RT‐qPCR system (ABI, MA) (Wang *et al*., 2023). Table S3 lists all primer information.

### *MdWRKY70L* , *MdSAG101* , *MdZAT12* and *MdMPK6/02G* overexpression or silencing in fruits

The transient overexpression vectors *MdWRKY70L*‐pCAMBIA2300, *MdSAG101*‐pCAMBIA2300, *MdZAT12*‐pCAMBIA2300, and *MdMPK6/02G*‐pCAMBIA1300 were constructed by cloning corresponding coding sequences (CDS) into either pCAMBIA2300 or pCAMBIA1300 vectors, while the silencing vectors *MdWRKY70L*‐pTRV2, *MdSAG101*‐pTRV2, *MdZAT12*‐pTRV2, and *MdMPK6/02G*‐pTRV2 were obtained by inserting fragments specific to *MdWRKY70L*, *MdSAG101*, *MdZAT12*, and *MdMPK6/02G* into pTRV2. The verified plasmids were introduced into *A*. *tumefaciens* strain GV3101 and used to infiltrate ‘Ruixue’ or ‘Fuji’ apples at 165 DAFB. Following 5 days in dark conditions, the peel surrounding the infiltration location was collected for phenotypic assessment and RT‐qPCR with primers listed in Tables S3 and S4.

### *MdWRKY70L* overexpression and knockout in ‘Orin’ calli

*MdWRKY70L* CDS was cloned into the pCAMBIA2300 vector for overexpression. CRISPR/Cas9 knockdown targets and corresponding primers (Table S4) for *MdWRKY70L* were selected using the website http://crispr.hzau.edu.cn/CRISPR2/. The target single‐guide RNA was cloned into pHSE401 and introduced into *A*. *tumefaciens* LBA4404 cells, which were kept at −80 °C until calli transformation.

### EMSA assay

*MdWRKY70L* CDS was cloned into pET32a‐His and transformed into *E. coli* BL21. The induced proteins were purified and stored at −80 °C. The potential MdWRKY70L‐binding sites in senescence‐related genes' promoter regions were analysed with PlantCARE software. Biotin‐labelled probes, unlabelled competitive probes, and mutant probes were designed for these sites. The binding specificity was confirmed using the LightShift Chemiluminescent EMSA kit (Thermo, Waltham, MA, USA). Table S4 lists all used primers.

### Dual‐LUC reporter analysis

The LUC analysis was executed as reported by Wang *et al*. (2023). *MdWRKY70L* CDS was inserted into the effector vector pGreenII 62‐SK driven by the CaMV35S promoter. *MdSAG101* and *MdZAT12 p*romoters were cloned into the reporter vector pGreenII 0800‐LUC. These vectors, along with the helper plasmid P19, were introduced into *A*. *tumefaciens* LBA4404 cells for transient expression in 4‐week‐old *Nicotiana benthamiana* leaves. The REN sequence in pGreenII 0800‐LUC, controlled by the 35S promoter, acted as the positive control. Firefly and Renilla luciferase activities were determined using the Infinite M200 (Tecan, Switzerland, Männedorf) with six replicates, and LUC activity 3 days post‐infiltration was visualized using an *in vivo* NightOwl II LB983 imaging system (Berthold Technologies, Germany, Bad Wildbad). Table S4 lists all used primers.

### ChIP‐PCR assay

The ChIP‐PCR assay was conducted as previously described (Wang *et al*., 2023). The transgenic calli harbouring MdWRKY70L fused to a GFP tag were prepared for the ChIP‐PCR assay. The ChIP experiment was carried out using an EZ CHIP 244 Chromatin Immunoprecipitation Kit (Upstate, Waltham, MA), following the manufacturer's instructions. The WT calli with detectable GFP tag were the negative control. PCR was performed to determine the DNA fragments using primers containing the specific binding regions in the *MdSAG101* and *MdZAT12* promoters. The primers used are listed in Table S4.

### Y2H assay

*MdMPK6/02G* CDS was cloned into pGBKT7 and co‐transformed with pGADT7 vector into Y2H yeast cells. Simultaneously, *MdWRKY70L* CDS was cloned into the pGADT7 vector. Y2H assays were conducted, as reported previously (Zhang *et al*., 2023). *MdMPK6/02G* and *MdWRKY70L* interaction was evaluated by observing yeast growth on tryptophan, leucine, histidine, and adenine‐deficient medium. Table S4 lists all used primers.

### BiFC assay

For the BiFC analysis, *MdMPK6/02G* and *MdWRKY70L* CDS were fused with the N‐terminal vector pSPYNE‐YFP and C‐terminal vector pSPYCE‐YFP, respectively. After transformation into *Agrobacterium* cells, they were co‐injected into tobacco leaves. The fluorescence signals, indicating protein–protein interaction, were observed under an ultra‐high‐resolution microscope 2 days post‐injection. Table S4 lists all used primers.

### Firefly LCI assay

For the LCI assay, *MdMPK6/02G* and *MdWRKY70L* CDS were cloned into vector pCAMBIA1300‐cLUC and pCAMBIA1300‐nLUC, respectively. After transformation into *Agrobacterium* cells, they were co‐injected into tobacco leaves. Fluorescence activity was detected *in vivo* using imaging techniques for better clarity. Table S4 lists all used primers.

### Pull‐down assay

*MdWRKY70L* and *MdMPK6/02G* CDS were cloned into pET‐32a (+) and pGEX‐4T‐1 and transformed into *E. coli* BL21 cells to produce His‐tagged and GST‐tagged fusion proteins, respectively. These proteins were purified using a commercial protein purification kit (Beyotime Biotechnology, Shanghai, China) and subjected to Western blotting using anti‐GST and anti‐His antibodies (Abmart, Shanghai, China). Table S4 lists all used primers.

### Protein phosphorylation detection

The assay was conducted in Beijing Bio‐Tech Pack Technology Company Ltd (Beijing, China, Haidian District). In detail, 10 μg proteins in 100 μL of 50 mmol/L NH^4^HCO^3^ were reduced with 10 mmol/L DTT for 1 h at 56 °C and incubated with 50 mmol/L IAM for 40 min in the dark. After that, proteins were digested at 37 °C for 4 h with 1% trypsin and 16 h with 2% trypsin. After desalting using a self‐packed column, peptides were dried at 45 °C using a vacuum centrifuge and re‐solubilized in 0.1% formic acid. After centrifugation at 16 000 ***g*** for 10 min at 4 °C, samples were subjected to mass spectrometry analysis for over 66 min using a 100 μm i.d. × 180 mm pre‐packed 3 μm Reprosil‐Pur 120 C18‐AQ column with 0.1% formic acid as mobile phase A and 0.1% formic acid in 80% ACN as mobile phase B at a flow rate of 600 nL/min.

### Validation of protein phosphorylation *in vitro*

*MdWRKY70L* phosphorylation at Ser199 was identified through IP/MS analysis. For further *in vitro* validation, the site was mutated to aspartic acid. MdWRKY70L and mutated MdWRKY70L (MdWRKY70L^S199^) and CAMdMPK6/02G were cloned into pGEX4T‐1‐GST, expressed in *E. coli* BL21 cells and purified, respectively. The purified MdWRKY70L and mutated MdWRKY70L^S199^ proteins were mixed with CAMdMPK6/02G, respectively, at a 1:5 ratio and incubated in kinase reaction buffer at 30 °C for 40 min. MdWRKY70L phosphorylation by MdMPK6/02G was assessed through Western blotting. Table S4 lists all used primers.

### Cell‐free protein degradation experiment

Purified GST‐tagged proteins were used as controls. CAMdMPK6/02G‐GST was reacted with MdWRKY70L‐His and MdWRKY70L^S199^‐His in an ATP‐containing kinase reaction buffer for 0, 20, 40, and 60 min. Western blot analysis was conducted to evaluate the degradation rate of MdWRKY70L.

### GUS activity analysis

The *MdSAG101/MdZAT12* promoter was inserted into the pCAMBIA1305‐GUS vector to generate the pro*MdSAG101/MdZAT12*::GUS vector. The empty promoter (Empty::GUS) as a negative control, using *Agrobacterium* transformation, 35S::CAMdMPK6/02G, 35S::MdWRKY70L, and 35S::MdWRKY70L^S199^ were co‐injected into tobacco leaves with the recombinant vectors for targeted injection. After co‐culture for 2 days, the leaves were stained for GUS and assayed for activity. Table S4 lists all used primers.

### Western blotting

Western blotting was executed as previously described (Wang *et al*., 2023) using anti‐GFP, anti‐His, anti‐GST, and anti‐phos antibodies from Abmart Medical Technology (Shanghai, China) Co., Ltd. Briefly, 0.1 g of transgenic calli were taken, and 500 μL of lysis buffer was added, followed by the addition of 5.0 μL each of protein inhibitors PMSF and cocktail. The mixture was ground on ice. Then, at 4 °C, it was centrifuged at 13 000 ***g*** for 10 min. A certain volume of the supernatant was taken and mixed with 5× loading buffer. After boiling in a water bath for 10 min, the prepared protein was placed on ice for gel electrophoresis. After the protein was transferred to a PVDF membrane, it was incubated with primary and secondary antibodies diluted 5000 times and 10 000 times, respectively, and then, the membrane was washed with the chemiluminescent working solution for imaging.

### Statistical analysis

All experiments were executed with three biological repeats. Data were processed using Microsoft Excel 2010, SigmaPlot 13 and Origin 2017 and compared using one‐way analysis of variance (ANOVA) and Student's *t‐*test using SPSS 24.0 (Armonk, USA), with *P* < 0.05 considered significant.

## Funding information

This study was sponsored by the Earmarked Fund for Modern Agro‐industry Technology Research System, China (CARS‐27); the National Key Research and Development Program of China (2023YFD2301000); the Major Science and Technology Projects in Shaanxi Province (2020zdzx03‐06‐02‐02); the Northwest A&F University Weinan Experimental Demonstration Station Construction Project (2024WNXNZX‐1); the Postdoctoral Fellowship Program of CPSF (GZC20232159); and the General Program of China Postdoctoral Science Foundation (2024M762645).

## Author contributions

Z.Z. and H.W. conceived the study. H.W., F.Y., S.Z., S.L., and Y.P. executed the experiments, provided reagents and materials, and analysed the data. H.W., S.Z., and Z.Z. prepared the paper.

## Competing interests

The authors declare no competing interests.

## Supporting information


**Table S1** Identification of phosphorylated peptides.
**Table S2** The proteins identified in a MdWRKY70L yeast two‐hybrid library interaction.
**Table S3** The primer sequences for RT‐qPCR.
**Table S4** Primers for transgene construction, CRISPR/Cas9‐based knockout, electrophoretic mobility shift assay (EMSA), luciferase assay (LUC), yeast two‐hybrid assay (Y2H), bimolecular fluorescence complementation assay (BIFC), luciferase complementation imaging (LCI), GUS, and CHIP‐PCR.

**Figure S1** Determination of antioxidant capacity and ROS enzyme activity in different parts of apple fruits during senescence.
**Figure S2** Identification of expression levels of the *MdWRKY70L* gene in different parts of fruit during development.
**Figure S3** Determination of antioxidant oxidase activity in apple and ‘Orin’ calli after instantaneous and stable transformation of *MdWRKY70L*.
**Figure S4** Senescence‐related gene expression levels in fruits at various stages.
**Figure S5** Senescence‐related gene expression levels after *MdWRKY70L* transfection into apple and ‘Orin’ calli.
**Figure S6** Acquisition and identification of ‘Orin’ calli with stable overexpression of *MdZAT12* and *MdSAG101* genes.
**Figure S7** Determination of antioxidant oxidase activity in ‘Orin’ calli after stable transformation of *MdZAT12* and *MdSAG101*.
**Figure S8** Determination of antioxidant oxidase activity after stable transformation of *MdZAT12* and *MdSAG101* into *MdWRKY70L* overexpression and knockout ‘Orin’ calli.
**Figure S9** Total ion flow chromatogram of stable transgenic *MdWRKY70L* calli.
**Figure S10** MdWRKY70L phosphorylation at Ser199 by MdMPK6/02G accelerated fruit senescence.
**Figure S11** Determination of antioxidant oxidase activity after instant infection with MdMPK6/02G and MdWRKY70L into apple fruits.

## Data Availability

All RNA‐seq data were submitted to https://www.ncbi.nlm.nih.gov/sar under accession number PRJNA861871. Other sequencing data can be downloaded from http://plants.ensembl.org/index.html under MD09G0105800 for *MdWRKY1*, MD13G0059600 for *MdWRKY3*, MD03G0162000 for *MdWRKY31*, MD03G0048200 for *MdWRKY24*, MD13G0134000 for *MdWRKY48*, MD05G0248800 for *MdWRKY65*, MD09G0202900 for *MdWRKY69*, MD01G0136400 for *MdWRKY70L*, MD13G0068300 for *MdWRKY72A*, MD13G0108800 for *MdWRKY75*, MD15G0034900 for *MdWRKY76*, MD09G0034000 for *MdSAG101*, MD14G0164000 for *MdEDS1*, MD12G0174000 for *MdCBP60F*, MD13G0103200 for *MdCYP76B6*, MD17G0093500 for *MdACO1*, MD14G0097100 for *MdACS1*, MD11G0144200 for *MdAAO1*, MD04G0166700 for *MdLOX1.5*, and MD07G0159300 for *MdZAT12*.
