# Supplementary material for: Phosphorylation of MdWRKY70L by MdMPK6/02G mediates reactive oxygen accumulation to regulate apple fruit senescence
Source: Plant Biotechnol J. 2025 Mar 24;23(6):2386–99. doi: 10.1111/pbi.70067 (PMC12120888; doi:10.1111/pbi.70067)
Supplement: Supplementary file 1 — Table S1 Identification of phosphorylated peptides. Table S2 The proteins identified in a MdWRKY70L yeast two‐hybrid library interaction. Table S3 The primer sequences for RT‐qPCR. Table S4 Primers for transgene construction, CRISPR/Cas9‐based knockout, electrophoretic mobility shift assay (EMSA), luciferase assay (LUC), yeast two‐hybrid assay (Y2H), bimolecular fluorescence complementation assay (BIFC), luciferase complementation imaging (LCI), GUS, and CHIP‐PCR. [file PBI-23-2386-s001.docx]

**Supplemental Table S1.** Identification of phosphorylated peptides based on LC-MS/MS.


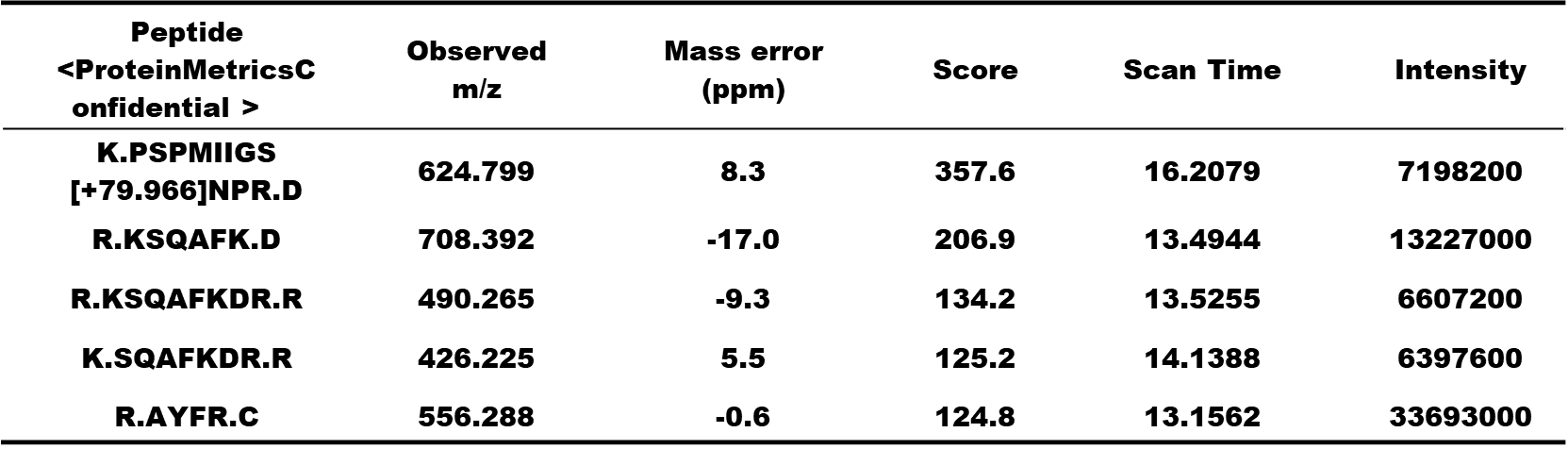


Note: Peptide represents the amino acid sequence identified to the peptide segment; m/z represents the mass-charge ratio of the peptide; Score represents for peptide score; Scan represents the mass spectrometry number for which the peptide has been identified; Intensity represents the abundance of the peptide; S,T,Y[+79.966]: Phospho (STY) phosphorylation occurs on serine, threonine and tyrosine with molecular weight of +79.966 Da.

**Supplemental Table S2.** The proteins identified in a MdWRKY70L yeast two-hybrid library interaction.

| Gene ID | Annotated Function |
| --- | --- |
| MD07G1009000 | ADP, ATP carrier protein 1, mitochondrial-like |
| MD13G1060200 | probable 6-phosphogluconolactonase 2 |
| MD04G1044600 | 60S ribosomal protein L3 |
| MD09G1178600 | protein TIFY 11B-like |
| MD12G1057600 | omega-6 fatty acid desaturase, endoplasmic reticulum isozyme 2 |
| MD02G1004000 | MAP protein kinase 6 |
| MD04G1030300 | chitinase-like protein 1 |
| MD17G1034800 | formate dehydrogenase, mitochondrial |
| MD15G1073300 | 3-oxo-Delta (4,5)-steroid 5-beta-reductase-like |
| MD07G1174700 | probable aquaporin PIP2-5 |
| MD15G1303500 | xyloglucan endotransglucosylase/hydrolase 6 |
| MD07G1215400 | plasma membrane intrinsic protein 1 |
| MD06G1015000 | S-adenosyl-L-methionine-dependent methyltransferases superfamily protein |
| MD00G1102700 | Heavy metal transport/detoxification superfamily protein |
| MD12G1231600 | copper/zinc superoxide dismutase 1 |
| MD08G1061500 | glycine decarboxylase P-protein 1 |

**Supplemental Table S3.** The primer sequences for RT-qPCR

| Gene name | Forward primer sequence | Reverse primer sequence |
| --- | --- | --- |
| *MdActin* | TGACCGAATGAGCAAGGAAATTACT | TACTCAGCTTTGGCAATCCACATC |
| *MdSAG101* | GGATAGTTATCACTGGACATTC | CAAGGGTGAACCGAAAGT |
| *MdEDS1* | TGGACCATCAGATGTTGTT | CCGACACTTCTCAAGGAA |
| *MdCBP60F* | CGGAGAATTGTGCGAGAG | AAGCCACATCCTCCAGAA |
| *MdCYP76B6* | ATTCCCACTAGGCTTCCA | ACTTGTCCGAGATGTAATGT |
| *MdACO1* | TTGGACACAGTGGAGAGG | GATGTTGGATTGAGGAAGATG |
| *MdACS1* | CCCACCAAGAACCCTAATG | TGGAGGCTTCTGGATTGT |
| *MdAAO1* | GGTGCTTGTGTTGTGCTA | GCCTTCGGATGTTGTAATTG |
| *MdLOX1.5* | AACGACTGGTCCAATGAA | TCAACGGTGATGTTCTGG |
| *MdYLS8* | GCTTCAGTTGCTGAGACA | CCGTGCCAAGATCAATCA |
| *MdNHL1* | GTCACTGTCTTCAACTTCAAC | GTAGATGCCGAGCTTGTC |
| *MdNPR1* | GGTGCCAAATCTTGAAGTAA | AGCCAATATACATCGGTGAA |
| *MdCBP60F* | CGGAGAATTGTGCGAGAG | AAGCCACATCCTCCAGAA |
| *MdSARD1* | TTAGGAGAGTGGTGAGTGA | AGTTGTAAGCTGGATTGTTC |
| *MdACC1* | ACATCAGAATTGCCGATCA | TGTGTTATCTCTGCCATCTC |
| *MdPDF1B* | TCTGAGAGCGAAGAACAAG | CTGAGAGCCCTATTCCATC |
| *MdZAT12* | CAATATGACTCCTCCGTCTC | TGGTCAGAACTATTGCTGTT |
| *MdWRKY1* | GATATGGTCGTTGATTCTGA | CGCCTTCTGACTTAATTCC |
| *MdWRKY3* | CATGTAACGTCCGTAAGC | CATTGTTGTTGCCTGTGTA |
| *MdWRKY31* | CGGTTCTCGATAGCCTTC | GACAACAATAGAGGTGATGG |
| *MdWRKY24* | TTGTATGGGCACGTAGGA | AGGCTCTTTAGCTGGGATA |
| *MdWRKY48* | TCGTCGTGGAGATATTACTT | GAAGAAGAGGAAGAAGAAGATG |
| *MdWRKY65* | AACACAAACTGGCTACTCA | TCCTCTTCTTCTGCTTCTAA |
| *MdWRKY69* | GCGGCATCAACAGTTGTA | GATTCCACCATCCTATTCCTAT |
| *MdWRKY70L* | AGATCGTGTCTTGGCGGAG | GCAGACTGGTGATCCTCAT |
| *MdWRKY72A* | TGGAGTTGATGAGGTCTTG | CTACGAAGGAAGCCACAA |
| *MdWRKY75* | ACCTTAAGAGCTAAGCACTT | TGAATTCCTGACACGTACAT |
| *MdWRKY76* | GTGTTCCTTCAAGTAGTATCG | TCAACTCTTCTGTCGCAAT |

**Supplemental Table S4.** The primer sequences for transgene, CRISPR/Cas9 knockout, EMSA, LUC, Y2H, BIFC, LCI, GUS and CHIP-PCR.

| Gene name | Forward primer sequence | Reverse primer sequence |
| --- | --- | --- |
| *MdZAT12*-W-box(hot probe) | CATTGATCTGGATTTCTGTGCTGACTCAGACTCATCGTGGATGCTT  (3`Biotin) | AAGCATCCACGATGAGTCTGAGTCAGCACAGAAATCCAGATCAATG  (3`Biotin) |
| *MdZAT12*-W-box(cold probe)  *MdZAT12*-W-box(mutant probe) | CATTGATCTGGATTTCTGTGCTGACTCAGACTCATCGTGGATGCTT  CATTGATCTGGATTTCTGTGCGGCCGCAGACTCATCGTGGATGCTT  (3`Biotin) | AAGCATCCACGATGAGTCTGAGTCAGCACAGAAATCCAGATCAATG  AAGCATCCACGATGAGTCTGCGGCCGCACAGAAATCCAGATCAATG  (3`Biotin) |
| *MdSAG101-W-box(hot probe)*  *MdSAG101-W-box(cold probe)*  *MdSAG101-W-box(mutant probe)* | TCTATATAAAGAGAGACATATTGACCATTCCGGTATCCTCTTCTCT  (3`Biotin)  TCTATATAAAGAGAGACATATTGACCATTCCGGTATCCTCTTCTCT  TCTATATAAAGAGAGACATATCGGCTATTCCGGTATCCTCTTCTCT  (3`Biotin) | AGAGAAGAGGATACCGGAATGGTCAATATGTCTCTCTTTATATAGA  (3`Biotin)  AGAGAAGAGGATACCGGAATGGTCAATATGTCTCTCTTTATATAGA  AGAGAAGAGGATACCGGAATAGCCGATATGTCTCTCTTTATATAGA  (3`Biotin) |
| *MdWRKY70L-YFP^C^*  *MdWRKY70L-YFP^N^*  *MdMPK6/02G-YFP^C^*  *MdMPK6/02G-YFP^N^*  *MdMPK6/02G-32a*  *MdMPK6/02G-PGEX4T*  *MdWRKY70L-32a*  *MdWRKY70L-PGEX4T*  *35S:MdWRKY70L-nLUC*  *35S:cLUC-MdMPK6/02G*  *P-MdWRKY70L*  *P-MdWRKY70L^S199^*  *QCJC-MdWRKY70L*  *QCJYJC-ID*  *MdSAG101-0800LUC*  *MdZAT12-0800LUC*  *MdSAG101(**pCAMBIA2300)*  *MdSAG101(pCAMBLA1300)*  *MdZAT12(pCAMBIA2300)*  *MdZAT12(pCAMBLA1300)*  *MdSAG101(pTRV2)*  *MdZAT12(pTRV2)*  *MdWRKY70L(pCAMBIA101)*  *MdMPK6/02G(pCAMBIA101)*  *MdWRKY70L(pTRV2)*  *MdWRKY70L-62SK*  *AD-MdWRKY70L*  *BD-MdWRKY70L*  *AD-MdMPK6/02G*  *BD-MdMPK6/02G*  *CA-MdMPK6/02G*  *MdZAT12*-CHIP  *MdSAG101*-CHIP  *MdZAT12*-GUS  *MdSAG101*-GUS  *MdWRKY70L-DT1-Bs*  *MdWRKY70L-DT2-Bs*  *MdWRKY70L-DT1-F0*  *MdWRKY70L-DT2-R0* | GCCTGGCGCGCCACTAGTGGATCCATGGGAACCAACCACAAGAGAC  GCCTGGCGCGCCACTAGTGGATCCATGGGAACCAACCACAAGAGAC  GCCTGGCGCGCCACTAGTGGATCCATGGAGGGAGGAGGGCGATCGG  GCCTGGCGCGCCACTAGTGGATCCATGGAGGGAGGAGGGCGATCGG  gccatggctgatatcggatccATGGAGGGAGGAGGGCGA  gatctggttccgcgtggatccATGGAGGGAGGAGGGCGA  gccatggctgatatcggatccATGGGAACCAACCACAAGAGAC  gatctggttccgcgtggatccATGGGAACCAACCACAAGAGAC  GAGCTCGGTACCCGGGATCCATGGGAACCAACCACAAGAGAC  TCCCGGGGCGGTACCATGGAGGGAGGAGGGCGATCGG  ATGGGAACCAACCACAAGAGAC  GATCATCGGCGATAATCCCAGGG  ATGGGAACCAACCACAAGAG  TGTCCCAGGATTAGAATGATTAGGC  GggccccccctcgaggtcgacACACGCAAACGTACAAAAGAAGAC  gggccccccctcgaggtcgacAACTAATTAAATAGTTGGTGGTGCATC  CGGGGGACGAGCTCGGTACCATGGTTTCGTTTACTCCCAAGAAC  gagaacacgggggacgagctcATGGTTTCGTTTACTCCCAAGAAC  CGGGGGACGAGCTCGGTACCATGAACATGAAGAGAAGCATCGCA  gagaacacgggggacgagctcATGAACATGAAGAGAAGCATCGCA  gtgagtaaggttaccgaattcCCCGCAACAGCGCAGCGCAA  gtgagtaaggttaccgaattcCGGAGGTCATCGAGCCAGCC  ttgatacatatgcccgtcgacATGGGAACCAACCACAAGAGAC  ttgatacatatgcccgtcgacATGGAGGGAGGAGGGCGA  gtgagtaaggttaccgaattcAACCCACTCACGTACCAAACCA  cgctctagaactagtggatccATGGGAACCAACCACAAGAGAC  gtaccagattacgctcatatgATGGGAACCAACCACAAGAGAC  tcagaggaggacctgcatatgATGGGAACCAACCACAAGAGAC  gtaccagattacgctcatatgATGGAGGGAGGAGGGCGA  tcagaggaggacctgcatatgATGGAGGGAGGAGGGCGATCGG  CCTCAGAGACTGGTTTTATGACTGCGTAT  GTTACCAATTAATTACAAAGCCC  CCTTTTGATTGGTGAGACAAAG  tatgaccatgattacgaattcAACTAATTAAATAGTTGGTGGTGCATC  tatgaccatgattacgaattcACACGCAAACGTACAAAAGAAGAC  ATATATGGTCTCGATTGGCTACATGAAGGCCAGAAGAGTT  TGGCTACATGAAGGCCAGAAGAGTTTTAGAGCTAGAAATAGC | GGGAGCGGTACCCTCGAGGTCGACTAGGAAACTCTCAAAATTAAAATCTTCC  GGGAGCGGTACCCTCGAGGTCGACTAGGAAACTCTCAAAATTAAAATCTTCC  GGGAGCGGTACCCTCGAGGTCGACCTGTCGCTGGTACTCGGGGTTAAAT  GGGAGCGGTACCCTCGAGGTCGACCTGTCGCTGGTACTCGGGGTTAAAT  gccatggctgatatcggatccATGGAGGGAGGAGGGCGA  ctcgagtcgacccgggaattcCTGTCGCTGGTACTCGGGG  ttgtcgacggagctcgaattcTAGGAAACTCTCAAAATTAAAATCTTCC  ctcgagtcgacccgggaattcTAGGAAACTCTCAAAATTAAAATCTTCC  CGAGATCTGGTCGACTAGGAAACTCTCAAAATTAAAATCTTCC  CTGCAGGTCGACCTGTCGCTGGTACTCGGGGTTAAAT  ATGGGAACCAACCACAAGAGAC  CCCTGGGATTATCGCCGATGATC  GTGATCCTCATGGCCATCA  GTTGATGGATCGAAAGAAGAGGGCT  CgctctagaactagtggatccCGCTGTTGATCGTGAAGCG  cgctctagaactagtggatccACACAAGAGTTTGTGTAAAATGAGGAT  CCATGGTGTCGACTCTAGATAGACCAGCAAGAACTGAGACCCC  cgcatagctaatctgggatccTAGACCAGCAAGAACTGAGACCCC  CGGGGGACGAGCTCGGTACCATGAACATGAAGAGAAGCATCGCA  cgcatagctaatctgggatccGAAAAACCCAACAAGTGGAGCTGC  gagacgcgtgagctcggtaccTAGACCAGCAAGAACTGAGACCCCATCGGC  gagacgcgtgagctcggtaccGAAAAACCCAACAAGTGGAGCTGCTTTCCC  tcagaattcggtacccccgggTAGGAAACTCTCAAAATTAAAATCTTCC  tcagaattcggtacccccgggCTGTCGCTGGTACTCGGGG  gagacgcgtgagctcggtaccCTCAAAATTAATAGACTTCACCACATAATC  gtcgacggtatcgataagcttTAGGAAACTCTCAAAATTAAAATCTTCC  cagctcgagctcgatggatccTAGGAAACTCTCAAAATTAAAATCTTCC  ccgctgcaggtcgacggatccTAGGAAACTCTCAAAATTAAAATCTTCC  cagctcgagctcgatggatccCTGTCGCTGGTACTCGGGG  ccgctgcaggtcgacggatccCTGTCGCTGGTACTCGGGG  ATACGCAGTCATAAAACCAGTCTCTGAGG  TACTGCAGAACTAGTCTGCAGTG  AAGGATCGGAGGAGTTTGAAG  caggtcgactctagaggatccACACAAGAGTTTGTGTAAAATGAGGAT  caggtcgactctagaggatccCGCTGTTGATCGTGAAGCG  ATTATTGGTCTCGAAACCCAAGACACGATCTCCGTACCAA  AACCCAAGACACGATCTCCGTACCAATCTCTTAGTCGACTCTAC |
|  |  |  |
