# Supplementary material for: Phosphorylation of MdWRKY70L by MdMPK6/02G mediates reactive oxygen accumulation to regulate apple fruit senescence
Source: Plant Biotechnol J. 2025 Mar 24;23(6):2386–99. doi: 10.1111/pbi.70067 (PMC12120888; doi:10.1111/pbi.70067)
Supplement: Supplementary file 2 — Figure S1 Determination of antioxidant capacity and ROS enzyme activity in different parts of apple fruits during senescence. Figure S2 Identification of expression levels of the MdWRKY70L gene in different parts of fruit during development. Figure S3 Determination of antioxidant oxidase activity in apple and ‘Orin’ calli after instantaneous and stable transformation of MdWRKY70L. Figure S4 Senescence‐related gene expression levels in fruits at various stages. Figure S5 Senescence‐related gene expression levels after MdWRKY70L transfection into apple and ‘Orin’ calli. Figure S6 Acquisition and identification of ‘Orin’ calli with stable overexpression of MdZAT12 and MdSAG101 genes. Figure S7 Determination of antioxidant oxidase activity in ‘Orin’ calli after stable transformation of MdZAT12 and MdSAG101. Figure S8 Determination of antioxidant oxidase activity after stable transformation of MdZAT12 and MdSAG101 into MdWRKY70L overexpression and knockout ‘Orin’ calli. Figure S9 Total ion flow chromatogram of stable transgenic MdWRKY70L calli. Figure S10 MdWRKY70L phosphorylation at Ser199 by MdMPK6/02G accelerated fruit senescence. Figure S11 Determination of antioxidant oxidase activity after instant infection with MdMPK6/02G and MdWRKY70L into apple fruits. [file PBI-23-2386-s002.docx]

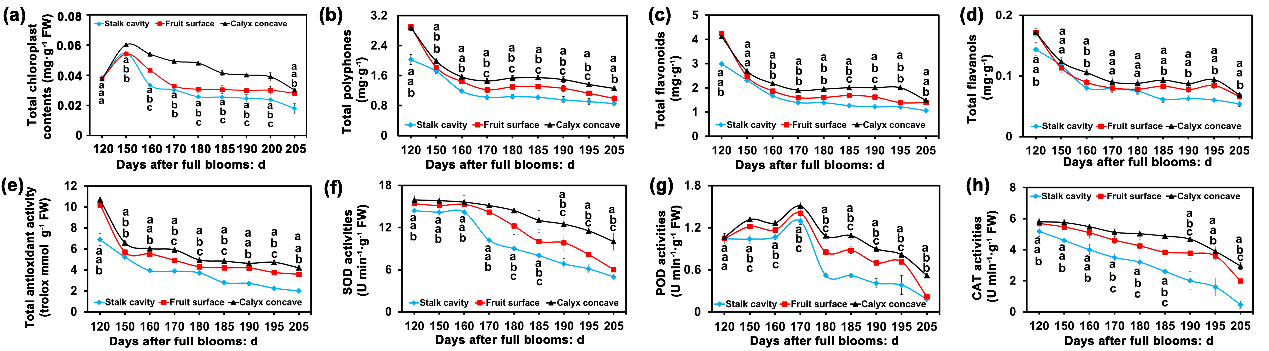


**Supplemental Figure** **S1.** Determination of antioxidant capacity and ROS enzyme activity in different parts of apple fruits during senescence. **(a)** Chlorophyll content. **(b)** Total polyphenols. **(c)** Total flavonoids. **(d)** Total flavanols. **(e)** Total antioxidant activity. **(f)** SOD activity. **(g)** POD activity. **(h)** CAT activity. Data shown are mean ± standard error with different letters denoting *P* < 0.05 (Student’s *t* test).

**Supplemental Figure** **S2.** Identification of expression levels of *MdWRKY70L* gene in different parts of fruit during development. Data shown are mean ± standard error with different letters denoting *P* < 0.05 (Student’s *t* test).

**Supplemental Figure S3.** Determination of antioxidant oxidase activity in apple and ‘Orin’ calli after instantaneous and stable transformation of *MdWRKY70L***. (a,d)** The activity of SOD. **(b,e)** The activity of POD. **(c,f)** The activity of CAT. Data shown are mean ± standard error with different letters denoting *P* < 0.05 (Student’s *t* test).


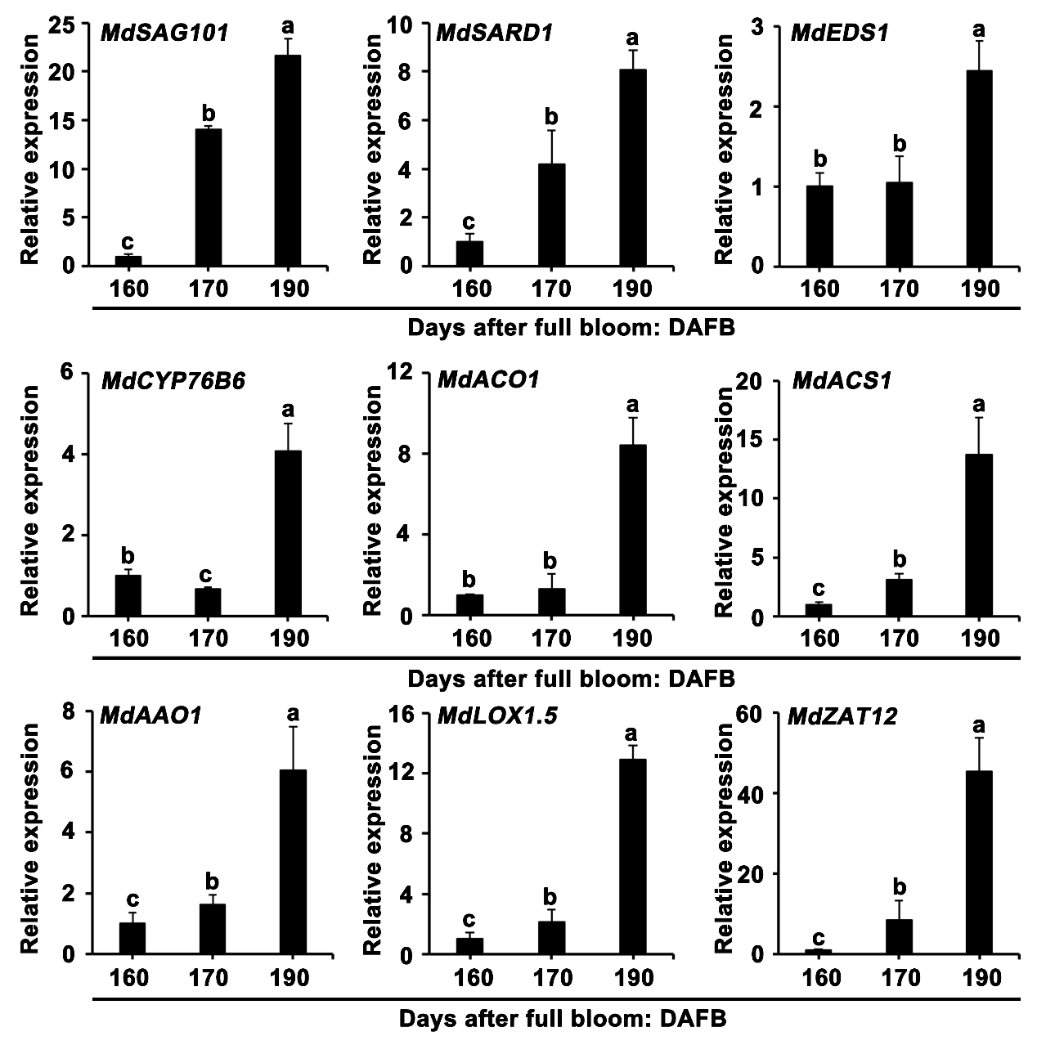


**Supplemental Figure** **S4.** Senescence-related gene expression levels in fruits at various stages. Different letters represent significant differences at *P* < 0.05 (Student’s *t* test).


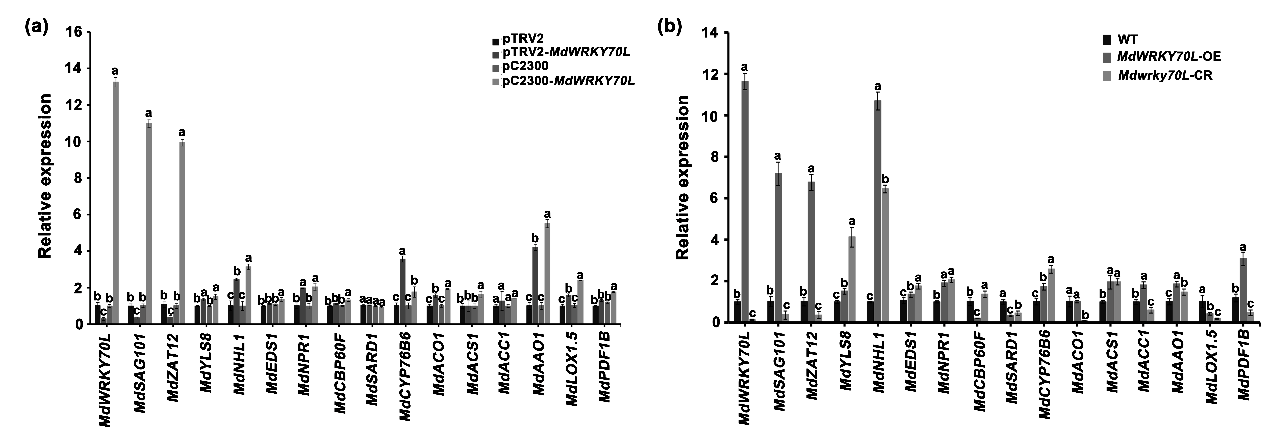


**Supplemental Figure S5.** Senescence-related gene expression levels after *MdWRKY70L* transfection into apple and ‘Orin’ calli. **(a)** Senescence-related gene expression levels after instant insertion of *MdWRKY70L* into apple. **(b)** Senescence-related gene expression levels after stable insertion of *MdWRKY70L* into ‘Orin’ calli. Data shown are mean ± standard error with different letters denoting *P* < 0.05 (Student’s *t* test).

**Supplemental Figure S6.** Acquisition and identification of ‘Orin’ calli with stable overexpression of *MdZAT12* and *MdSAG101* genes. **(a,d)** Phenotype of ‘Orin’ calli with stable overexpression of *MdZAT12* and *MdSAG101* genes. **(b,e)** The transgenic materials were identified by RT-qPCR. Data shown are mean ± standard error with different letters denoting *P* < 0.05 (Student’s *t* test). **(c,f)** Western blotting technique was used to identify transgenic materials.

**Supplemental Figure S7.** Determination of antioxidant oxidase activity in ‘Orin’ calli after stable transformation of *MdZAT12* and *MdSAG101***.** The activity of SOD **(a)**, POD **(b)** and CAT **(c)**. Data shown are mean ± standard error with different letters denoting *P* < 0.05 (Student’s *t* test).

**Supplemental Figure S8.** Determination of antioxidant oxidase activity after stable transformation of *MdZAT12* and *MdSAG101* into *MdWRKY70L* overexpression and knockout ‘Orin’ calli. The activity of SOD **(a)**, POD **(b)** and CAT **(c)**. Data shown are mean ± standard error with different letters denoting *P* < 0.05 (Student’s *t* test).


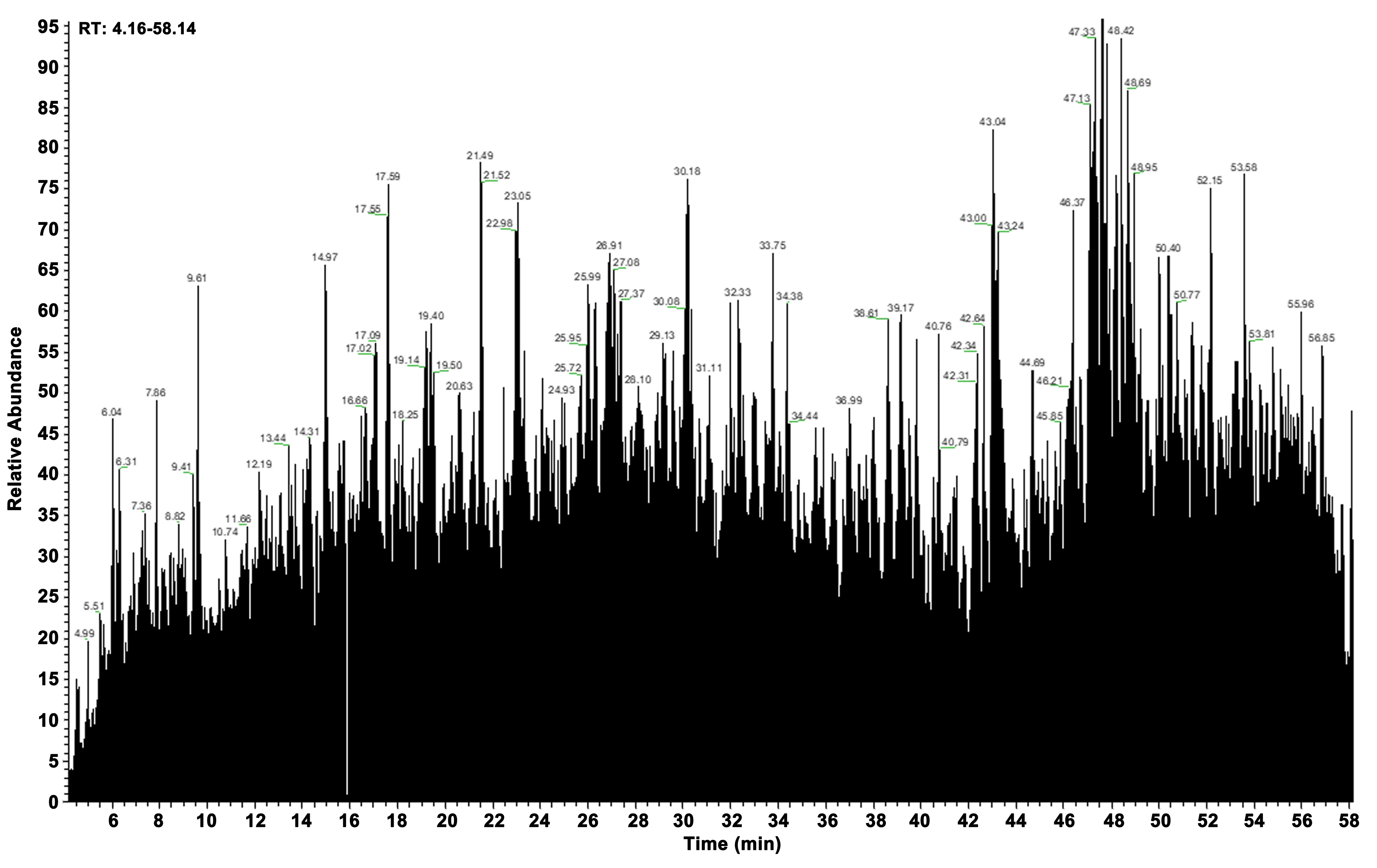


**Supplemental Figure S9.** Total ion flow chromatogram of stable transgenic *MdWRKY70L* calli.


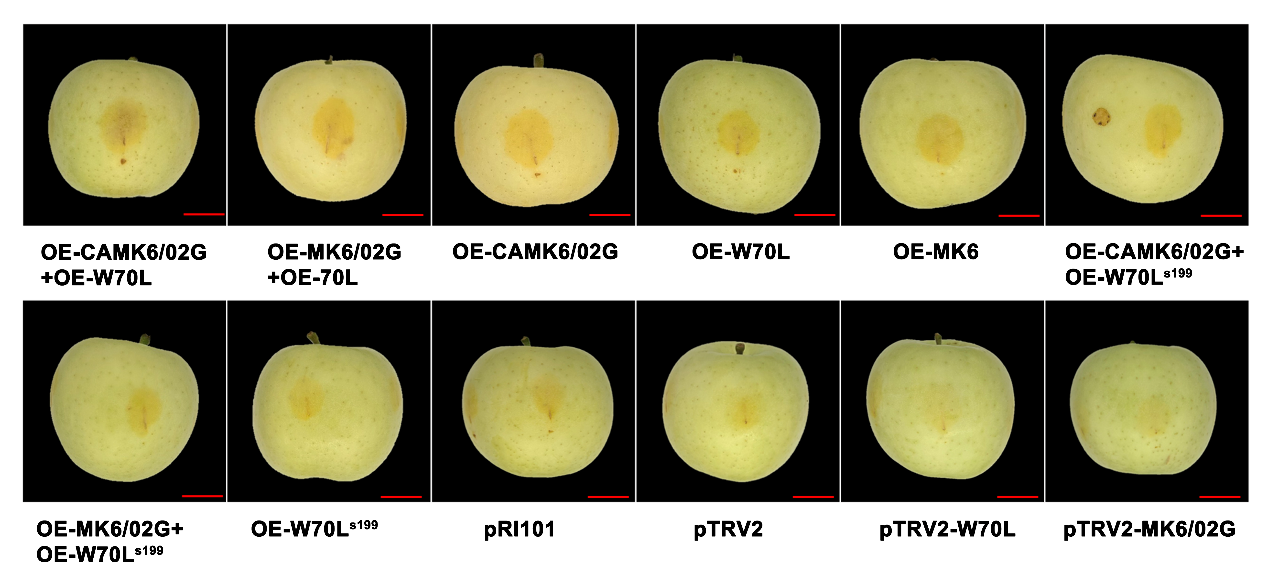


**Supplemental Figure S10.** MdWRKY70L phosphorylation at Ser199 by MdMPK6/02G accelerated fruit senescence. Scale bar = 2 cm. Apple images were digitally processed for comparison.

**Supplemental Figure S11.** Determination of antioxidant oxidase activity after instant infection with MdMPK6/02G and MdWRKY70L into apple fruits. The activity of SOD **(a)**, POD **(b)** and CAT **(c)**. Data shown are mean ± standard error with different letters denoting *P* < 0.05 (Student’s *t* test).
